# Protocol for regional implementation of collaborative lung function testing

**DOI:** 10.1038/npjpcrm.2016.24

**Published:** 2016-06-02

**Authors:** Claudia Vargas, Felip Burgos, Isaac Cano, Isabel Blanco, Pere Caminal, Joan Escarrabill, Carles Gallego, Ma Antonia Llauger, Felip Miralles, Oscar Solans, Montserrat Vallverdú, Filip Velickovski, Josep Roca

**Affiliations:** 1 Hospital Clinic de Barcelona, Institut d’Investigacions Biomèdiques August Pi i Sunyer (IDIBAPS), Universitat de Barcelona, Barcelona, Spain; 2 Center for Biomedical Network Research in Respiratory Diseases (CIBERES), Barcelona, Spain; 3 Centre de Recerca en Enginyeria Biomèdica (CREB-UPC), Universitat Politècnica de Catalunya, Barcelona, Spain; 4 Pla Director de les Malalties de l’Aparell Respiratori (PDMAR), Departament de Salut de Catalunya, Barcelona, Spain; 5 REDISSEC. Red de Investigación en Servicios de Salud en Enfermedades Crónicas, Madrid, Spain; 6 Oficina d’Estàndards i Interoperabilitat—Fundació TicSalut, Departament de Salut, Barcelona, Spain; 7 Institut Català de la Salut (ICS). Generalitat de Catalunya, Catalunya, Spain; 8 Eurecat. Technological Center of Catalonia, Catalunya, Spain

## Background

The potential of forced spirometry (FS) testing for diagnosis, monitoring and management of chronic respiratory patients is well established^1–3^ such that FS is a pivotal test in both respiratory medicine and primary care. Moreover, it also shows potential in the informal care scenario: that is, in pharmacy offices for case-finding purposes^4,5^ and for self-management in selected patients.^6,7^

We acknowledge that well-designed studies^8^ have failed to show practical benefits of FS for asthma and COPD diagnosis and management in primary care. However, it has been demonstrated that historical limitations for extensive use of FS in primary care, because of suboptimal quality of testing, can be overcome by off-line remote support by specialised professionals.^9,10^ Large-scale deployment of this type of setting has generated evidence of cost-effectiveness.^11,12^

**Figure 1:**
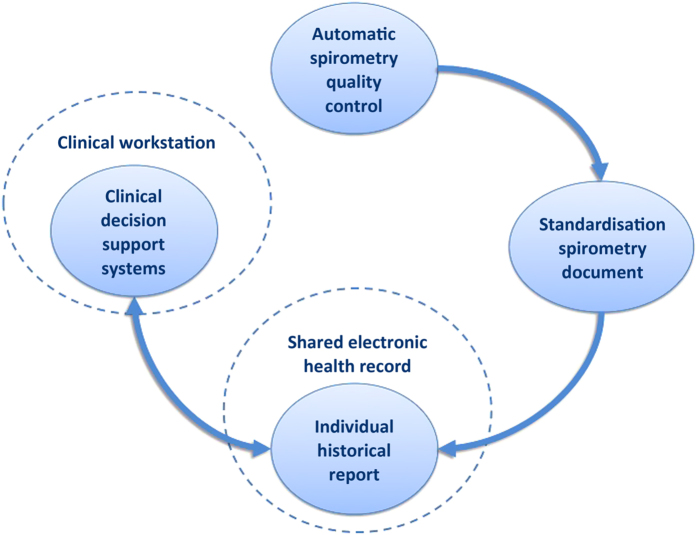
Four pivotal components (circles with light-blue background) for regional deployment of the collaborative FS program across health-care tiers. Dotted-line empty circles illustrate underlying health information systems.

All the above elements^13,14^ allow us to identify and develop^9,15–18^ the four pivotal components needed for regional deployment in Catalonia (ES; 7.5 million inhabitants) of a collaborative FS program across health-care tiers. The four core components of the FS program are illustrated in Figure 1. The underlying hypothesis is that the proposed FS program facilitates the transference of diagnostic testing from specialised care to non-specialised primary care professionals, which may generate significant health-care efficiencies and provide valuable information on longitudinal changes of lung function either spontaneously or because of interventions.

The current protocol describes the roadmap for scale-up of the Catalan collaborative FS program based on the existing regional interoperability setting among health-care providers, which has two principal components, namely, (i) the regional shared electronic health-care record^19^ allowing health information exchange among providers, and (ii) the personal health folder^20^ that facilitates citizen's accessibility to his/her health-care information.

## Aims

The current protocol describes the elements for the deployment of an innovative collaborative FS testing program across health-care tiers in Catalonia. The roadmap for deployment of the FS program has three well-defined actions. The first is implementation of the program in three health-care sectors during the first semester of 2016. Thereafter, deployment of the program throughout the entire Catalan region will be completed by the end of the year. Third, the research is paying specific attention to two distinctive transferability aspects: that is, adaptability of the FS program to sites having different interoperability schemes or no interoperability at all, and generalisation of this type of program to other respiratory (i.e., home-based sleep studies) and non-respiratory (i.e., electrocardiogram) testing modalities. Moreover, the deployment process of the FS program will also cover the needs for data analytics allowing longitudinal assessment of lung function changes with relevant implications in future personalised patient management.^21^

## Methods

The FS program emerges from a series of studies reporting on technological solutions for each of the main components illustrated in Figure 1.^9,16–18^ Their articulated application covers unmet needs for collaborative FS testing. The studies were initiated within the EU project NEXES,^22,23^ and specific parts of the overall setting have already been successfully evaluated in the Basque Country.^11,12^

### The setting and study design

The current protocol has been designed as part of the regional deployment of integrated care services in Catalonia.^22,23^ It consists of the two lines of activity ultimately aiming at (i) regional adoption of the FS program and (ii) generalisation of the approach to other areas, as well as to other testing procedures (i.e., home-based sleep studies, electrocardiogram and so on). The research was approved by the Ethical Committee of the Hospital Clínic de Barcelona, and it has been registered at ClinicalTrials.gov Id: NCT02592928.

### Program deployment

During the initial 6 months, the protocol will include three health-care sectors: Lleida (366k inhabitants and 22 Primary Care Centers, PCC), Vic (157k inhabitants and 11 PCC) and Atenció Integral en Salut Barcelona-Esquerra (520k inhabitants and 19 PCC, respectively) following a Plan-Do-Study-Act (PDSA) methodology.^24^ The main purpose of the first PDSA cycle (January–March 2016) including a total of three PCC, one in each health-care sector, is to ensure full functionality of the setting (Figure 1). Immediately thereafter, the FS program will be progressively deployed to all 52 PCC in the three health-care sectors in a second 3-month PDSA cycle that will be completed by mid-2016. FS testing will be prescribed by the attending general practitioner following standard criteria, and it will be carried out by primary care nurses.

The deployment of the program to the entire region (369 PCC) will be completed, with no discontinuation, within 2016. The outcomes of the assessment carried out during each PDSA cycle will contribute to identify key performance indicators (KPI) that are helpful in modulating the long-term assessment strategy of the FS program beyond the deployment phase.

### Transferability and data analytics

A relevant line of activity will be devoted to analysis of transferability and proposals for data analytics to be completed in parallel with deployment of the FS program. The transferability analysis will encompass two distinctive areas: (i) identification of deployment strategies for regions with limited or no interoperability architecture at regional level, and (ii) evaluation of the potential for transferring the characteristics of the program to other testing procedures, such as home-based sleep studies and/or electrocardiogram.

### Service workflow and ICT support

The section describes the technological support required to cover the four components of the FS program, as depicted in Figure 1: (i) enhanced automatic FS quality assessment; (ii) accessibility to standardised (and quality-labelled) FS testing information across health-care tiers; (iii) generation of an individual FS report including historical results from a given patient; and (iv) clinical decision support systems in the clinical workstation of primary care professionals, facilitating accessibility to the patient FS historical report, as well as access to off-line remote support by specialised professionals, upon request. A high-level description of the proposed logical workflow is presented and described (Supplementary Figure S1) in the online Supplementary Material.

### Roadmap for deployment and methodologies for assessment

The current status of the FS program shows a rather mature scenario.^25^ The deployment process will follow a PDSA methodology^24^ including iterations with different stakeholders, namely, primary care professionals (nurses, general practitioners), specialised professionals in respiratory medicine (doctors, allied health professionals), equipment manufacturers, information and communication technologists and so on. Regional adoption of the FS program is being stimulated by establishment of financial incentives to health-care providers to foster transfer of standardised FS testing into the shared EHR at the regional level (Supplementary Figure S1, steps 3 and 4). Moreover, the deployment of the FS program is aligned with different innovation initiatives implementing ICT-supported integrated care services in Catalonia. Different pilot experiences^22,23^ have contributed to identify four sequential milestones described in the online Supplementary Material.

### Capacity for generalisation to other diagnostic modalities

The protocol is expected to be the basis for future deployment of collaborative programs for many other respiratory and non-respiratory testing procedures. This is so, because the main components of the FS-specific service workflow and supporting health information systems are conceptually designed to be extrapolated to collaborative scenarios wherein care coordination and telehealth are key features facilitating efficient transfer of diagnostic tools from specialised and community care. For example, the guide for standardisation of spirometry documents (CDA) can be adapted to other diagnostic modalities (i.e., home-based sleep studies, electrocardiogram and so on) after reaching consensus among key opinion leaders and scientific societies for each specific diagnostics area.

## Data analytics

The large number of standardised FS documents, including quality certification, stored into the system should open avenues for privacy preserving data mining. For instance, longitudinal evolution of lung function decline^21,26^ may constitute a key element for enhanced clinical risk assessment and stratification. Moreover, analysis of variability could provide novel approaches for assessment of bronchial hyper-responsiveness. Effects of interventions on longitudinal evolution of lung function results may provide new insights on efficacy of clinical actions.

## Discussion

There is evidence that deployment of integrated care services for chronic patients can generate health-care efficiencies^22,27^ that are partly explained by the transfer of service complexities from specialised care to the community. In this scenario, availability of high-quality testing with a collaborative approach constitutes a relevant component of the chronic care model that fosters optimisation of health-care outcomes.

The current protocol shows the articulation of different technological developments^9,16–18^ carried out and validated in Catalonia during the last years in order to generate a collaborative ecosystem for diagnostic purposes across health-care tiers having FS testing as a use case. The research indicates that the Catalan FS program has high potential for transferability to other geographical areas despite having different interoperability schemes, as well as to other respiratory and non-respiratory testing procedures (see online Supplementary Material). Specifically, the report describes the strategy for scaling up the collaborative service at the regional level. Moreover, the protocol generated a proposal for long-term assessment of adoption of the FS program (Supplementary Table S2), beyond the initial deployment phase.

### Expected impact and future developments

Data analytics from the regional deployment of the collaborative FS program may generate immediate positive impacts on several areas, namely, (i) sustained increase of high-quality FS testing in primary care; (ii) collaborative environment across health-care tiers; (iii) enhanced professional development of health professionals; (iv) better diagnostic ascertainment for respiratory diseases; (v) reduction of unnecessary test duplication; and (vi) facilitation of coordinated interactions with FS testing carried out in the informal care scenario: that is, pharmacy offices for COPD case-finding purposes and home-based FS testing in selected patients (i.e., severe asthma refractory to treatment).^4,5^

## References

[bib1] Halbert, R. J. et al. Global burden of COPD: systematic review and meta-analysis. Eur. Respir. J. 28, 523–532 (2006). 16611654 10.1183/09031936.06.00124605

[bib2] Murray, C. J. & Lopez, A. D. Measuring the global burden of disease. N. Engl. J. Med. 369, 448–457 (2013). 23902484 10.1056/NEJMra1201534

[bib3] Jones, R. C. et al. Opportunities to diagnose chronic obstructive pulmonary disease in routine care in the UK: a retrospective study of a clinical cohort. Lancet Respir. Med. 2, 267–276 (2014). 24717623 10.1016/S2213-2600(14)70008-6

[bib4] Castillo, D. et al. COPD case finding by spirometry in high-risk customers of urban community pharmacies: a pilot study. Respir. Med. 103, 839–845 (2009). 19200706 10.1016/j.rmed.2008.12.022

[bib5] Castillo, D. et al. Airflow obstruction case finding in community-pharmacies: a novel strategy to reduce COPD underdiagnosis. Respir. Med. 109, 475–482 (2015). 25754101 10.1016/j.rmed.2015.02.009

[bib6] Finkelstein, S. M. et al. Monitoring progress after lung transplantation from home-patient adherence. J. Med. Eng. Technol. 20, 203–210 (1996). 9029392 10.3109/03091909609008999

[bib7] Magnussen, H. et al. Withdrawal of inhaled glucocorticoids and exacerbations of COPD. N. Engl. J. Med. 371, 1285–1294 (2014). 25196117 10.1056/NEJMoa1407154

[bib8] Lusuardi, M. et al. A randomized controlled trial on office spirometry in asthma and COPD in standard general practice: data from spirometry in Asthma and COPD: a comparative evaluation Italian study. Chest 129, 844–852 (2006). 16608929 10.1378/chest.129.4.844

[bib9] Burgos, F. et al. Telemedicine enhances quality of forced spirometry in primary care. Eur. Respir. J. 39, 1313–1318 (2012). 22075488 10.1183/09031936.00168010

[bib10] Muller-Brandes, C. et al. LUNOKID: can numerical American Thoracic Society/European Respiratory Society quality criteria replace visual inspection of spirometry? Eur. Respir. J. 43, 1347–1356 (2014). 24232698 10.1183/09031936.00058813

[bib11] Marina, N. et al. Economic Assessment and Budgetary Impact of a Telemedicine Procedure and Spirometry Quality Control in the Primary Care Setting. Arch. Bronconeumol. 52, 24–28 (2015). 25912937 10.1016/j.arbres.2015.02.012

[bib12] Marina, M. N. et al. Telemedicine spirometry training and quality assurance program in primary care centers of a public health system. Telemed. J. E Health 20, 388–392 (2014). 24476193 10.1089/tmj.2013.0111

[bib13] Llauger, M. A. et al. Accesibility and use of spirometry in primary care centers in Catalonia. Aten. Primaria 46, 298–306 (2014). 24768654 10.1016/j.aprim.2013.12.012 PMC6983645

[bib14] Roger, N. et al. Survey about the use of lung function testing in public hospitals in Catalonia in 2009. Arch. Bronconeumol. 49, 371–377 (2013). 23414603 10.1016/j.arbres.2012.12.006

[bib15] Burgos, F. Impact of Information and Communication Technologies on remote testing: Forced Spirometry as a user case (PhD Thesis, Univ. of Barcelona, 2013).

[bib16] Burgos, F. et al. Clinical decision support system to enhance quality control of spirometry using information and communication technologies. JMIR Med. Inform. 2, e29 (2014). 25600957 10.2196/medinform.3179 PMC4288080

[bib17] Melia, U. et al. Algorithm for automatic forced spirometry quality assessment: technological developments. PLoS ONE 9, e116238 (2014). 25551213 10.1371/journal.pone.0116238 PMC4281176

[bib18] Salas, T. et al. Technical requirements of spirometers in the strategy for guaranteeing the access to quality spirometry. Arch. Bronconeumol. 47, 466–469 (2011). 21821333 10.1016/j.arbres.2011.06.005

[bib19] Marimon-Sunol, S., Rovira-Barbera, M., Acedo-Anta, M., Nozal-Baldajos, M. A. & Guanyabens-Calvet, J. Shared electronic health record in Catalonia, Spain. Med. Clin. (Barc) 134(Suppl 1): 45–48 (2010). 10.1016/S0025-7753(10)70009-9 20211353

[bib20] Saigi, F., Cerda, C. I., Guanyabens, C. J. & Carrau, V. E. Personal health records: the case of the Personal Health Folder of Catalonia (Spain). Gac. Sanit. 26, 582–584 (2012). 22554458 10.1016/j.gaceta.2012.03.005

[bib21] Lange, P. et al. Lung-function trajectories leading to chronic obstructive pulmonary disease. N. Engl. J. Med. 373, 111–122 (2015). 26154786 10.1056/NEJMoa1411532

[bib22] Cano, I. et al. An adaptive case management system to support integrated care services: lessons learned from the NEXES project. J. Biomed. Inform. 55, 11–22 (2015). 25796455 10.1016/j.jbi.2015.02.011

[bib23] Hernandez, C. et al. Integrated care services: lessons learned from the deployment of the NEXES project. Int. J. Integr. Care 15, e006 (2015). 26034465 10.5334/ijic.2018 PMC4447233

[bib24] Taylor, M. J. et al. Systematic review of the application of the plan-do-study-act method to improve quality in healthcare. BMJ Qual. Saf. 23, 290–298 (2014). 10.1136/bmjqs-2013-001862 PMC3963536 24025320

[bib25] EIP-AHA, European Scaling-up Strategy in Active and Healthy Ageing. Available at http://ec.europa.eu/research/innovation-union/pdf/active-healthy-ageing/scaling_up_strategy.pdf (2014).

[bib26] Kohansal, R. et al. The natural history of chronic airflow obstruction revisited: an analysis of the Framingham offspring cohort. Am. J. Respir. Crit. Care Med. 180, 3–10 (2009). 19342411 10.1164/rccm.200901-0047OC

[bib27] ACT—Advancing Care Coordination and Telehealth deployment (DG Sanco/Health 20121209). Available at http://www.act-programme.eu/ (2015).

