# Supplementary material for: Protocol for regional implementation of collaborative lung function testing
Source: NPJ Prim Care Respir Med. 2016 Jun 2;26:16024–. doi: 10.1038/npjpcrm.2016.24 (PMC4890075; doi:10.1038/npjpcrm.2016.24)
Supplement: Supplementary Information [file npjpcrm201624-s1.doc]

**Supplementary Information**

# Protocol for Regional Implementation of Collaborative Lung Function Testing

Claudia Vargas et al.

**Table of contents:**

**Section 1 – Detailed description of the proposed logical workflow of the deployment**

- Automatic FS quality assessment
- Structured spirometry document
- Individual historical report
- Clinical decision support systems

**Roadmap for deployment and methodologies for assessment**

- Software development and user validation
- Deployment in the three healthcare sectors
- Scale-up of the FS program in the entire region
- Enhanced automatic quality control

**Transferability to other geographical areas**

- Spirometry quality control
- Standardized spirometry document
- Individual historical report
- Clinical decision support system (CDSS)

**Section 2 – Forced spirometry report and software (Java) prototype for the creation of the document**

**Section 3. Assessment of the deployment of Forced Spirometry program (Table 1S) and long-term evaluation of the program beyond the deployment phase (Table 2S)**

**Section 4. Standardized FS testing information - Implementation guide using a Clinical Document Architecture (CDA)**

The document includes four sections with relevant pieces of information complementing the text of the main manuscript.

**Section 1 – Detailed description of the deployment**

Firstly, **Figure 1S** present the description of the proposed logical workflow, which aims to generate two main outcomes, namely: i) An individual FS report, including quality-labelled results of historical testing available in the shared electronic care record (blue-coloured steps 1 to 5); and, ii) Clinical decision support systems providing smart FS testing accessibility in the clinical workstation (green coloured step 6), as well as triggering an external process for remote off-line consultation to specialized care for interpretation or diagnostic purposes (green coloured step 7)

Automatic FS quality assessment

The characteristics of the equipment for FS measurements and the recommendations for FS testing, including quality assessment, are both highly standardized by international guidelines^1;2^. However, it is acknowledged that the transfer of FS testing to non-specialized professionals in the community constitutes a challenge because poor quality of FS testing may generate misdiagnosis^3^. In order to enhance acceptability of the FS manoeuvres, the program (**Figure 1S**, **step 2**) includes an automatic enhanced algorithm for FS quality assessment^4;5^ following standards recommended by ATS/ERS (American Thoracic Society/European Respiratory Society) for FS testing^2^.

Structured spirometry document

Once the FS testing is performed, **step 3** considers exporting both the raw spirometric data, including all FS curves, and the FS report to the shared electronic health record at a regional level. Firstly, the raw spirometric data will be structured in accordance with the FS implementation guide complying with an HL7 (Health Level 7 International) standard. The English translation of the FS implementation guide using a CDA (Clinical Document Architecture), a standard intended to specify the encoding, structure and semantics of clinical documents for exchange^6^, is available in the on-line supplementary material.

**Step 4** in the logic workflow considers the storage of the structured spirometric data and the FS report both at the provider’s electronic health record (EHR) and at the shared electronic health record at regional level (HC^3^ in Catalonia)^7^. The latter will ensure broader accessibility of the FS testing report, irrespective of the healthcare provider and health level wherein testing was performed. Worth to highlight the potential for big data analytics of a repository of structured spirometry documents, as described below.

Individual historical report

**Step 5** consists on generating a Portable Document Format (PDF) version of the FS report (**Figure 1S**) following a consensus layout that includes the results of the last FS testing together with summary information on past spirometric results stored in the local or shared electronic health record. The characteristics of the software developed in order to generate the PDF version of the FS report is reported in the on-line supplementary material*.*

Clinical decision support systems

The accessibility to the individual historical report generated at the end of the previous process (**step 5**) requires a close interaction between the local, or shared, electronic health record and the clinical workstations (**step 6**). A clinical decision support system (CDSS) will advise health professionals about the readiness of a new FS testing report when, in a given patient, a respiratory diagnosis is being considered. Moreover, at this stage, a clinician may optionally trigger an external off-line consultation to a respiratory specialist (**step 7**) for clinical support in the interpretation of the FS testing results. To this end, the clinical workstation facilitates initiation of the remote consultation while providing access to the corresponding FS testing report to the two professionals interacting in the consultation.

**Roadmap for deployment and methodologies for assessment**

Different pilot experiences^8;9^ have contributed to identify four sequential milestones indicated below:

*Software development and user validation –* The four initial steps indicated in **Figure 1S** are already operational. Two additional pieces of software corresponding to: v) generation of the individual historical report using information stored in the shared EHR (**step 5**), as described in the on-line supplementary material; and, vi) CDSS supporting clinical interactions (**steps 6** and **7**) are being completed and validated by end-users within 2015.

*Deployment in the three healthcare sectors -* The first PDSA cycle will be undertaken in one primary care unit from each of the three initial healthcare sectors with two primary goals: i) to confirm that **steps 3** to **7** (**Figure 1S**) are fully operational; and, ii) to assess acceptability of the entire process by end-users; that is, primary care physicians and nurses. It will include analysis of acceptability of proposed indicators (**Table 1S**, Section 3*in the current document* for systematic evaluation of the deployment of the FS program, Thereafter, the two principal milestones of the second PDSA cycle will be: i) to achieve full deployment of the FS program in the fifty-two primary care units of the three healthcare sectors; and, ii) to collect the indicators indicated in **Table 1S**.

*Scale-up of the FS program in the entire region –* The three milestones to be achieved by the end of 2016 are: i) to complete implementation of the FS program in all primary care units (n=369) across the region; ii) to generate a final report assessing the deployment process based on the set of indicators depicted in **Table 1S**; and, finally, iii) to consolidate a new set of indicators for long-term monitoring of adoption beyond 2016 to analyze both sustainability and impact on healthcare, as proposed in **Table 2S**.

*Enhanced automatic quality control –* It has been identified that optimal performance of the algorithm for automatic quality control of FS testing^5^ will be achieved through its incorporation into the spirometer, either build-up into the original spirometer software or being remotely incorporated into the equipment, as part of the Internet of Things network. The two possibilities are currently being jointly analysed with spirometer manufacturers in order to achieve an optimal performance of **step 2** (**Figure 1S**) within the deployment time frame.

**Transferability to other geographical areas**

The transferability of the FS program to other geographical areas with different or no interoperability schemes is analysed for each of the four core components displayed in **Figure 1**.

*Spirometry quality control* - The module for automated quality assessment of spirometric maneuvres has been extensively reported^4;5^. Both module inputs and outputs are encoded within an agreed structured message that complies with the HL7 Virtual Medical Record (vMR). The version of the vMR standard that is used is specified by "HL7 V3 DAM: vMR-CDS, Release 1, April 2012", and it is available from the HL7 web site and in the on-line supplementary material. These characteristics ensure the capacity of the module to be adopted by manufacturers.

*Standardized spirometry document -* The Catalan healthcare standards and interoperability office (TicSalut), currently leading HL7 Spain, has defined a specific guide for standardization of FS documents (see on-line supplementary material); that is, generation of CDA ensuring transferability to other healthcare networks.

*Individual historical report -* **Figure 1S** (**step 4**) indicates storage of the structured FS document either in a local or shared electronic health record which will determine the potential range of healthcare suppliers and levels of care beneficiaries of the service. A real barrier for the transferability of the individual historical report will be the complete lack of an in-place electronic health record.

*Clinical decision support system (CDSS) -* The service workflow considers two main components of the CDSS (**Figure 1S**) facilitating access to the patient FS historical report and to off-line remote support from specialized professionals, upon request. The former CDSS component will be delivered as a web service, so that it could potentially be integrated with any corporate-specific clinical workstation. The latter CDSS component is proposed to be handled independently from the service workflow, so that any remote consultation service could be used, if in place.

Secondly, **Figure 2S** depicts the consensus format for the individual historical report generated within the shared electronic care record, as indicated in **Figures 1** and **1S**, **step 5.** The individual historical report will be displayed in the clinical workstation by the CDSS. The report includes complete information on the last FS testing together with data on lung function evolution over time with appropriate information on quality testing for each of the historical studies. Section 2 of the current document also includes a description of the basics of the software developed in order to generate the individual historical report (**Figure 2S**).

Thirdly, **Table 1S** indicates the main variables to be evaluated at the end of each PDSA cycle following the Method for Assessment of Telemedicine Applications (MAST)^10^, as reported in the main manuscript. The results of the assessment throughout the on-year deployment phase, 2016, will contribute to shape the final format of **Table 2S**. The table contains the main variables for automatic tracking of the program beyond the deployment phase, for 2017 and beyond. The aim is to assess maturity of the implementation and impact on selected healthcare outcomes.

The third element enclosed in the current document, section 3, is the English translation of the FS implementation guide using a CDA (Clinical Document Architecture). The CDA is a standard intended to specify the encoding, structure and semantics of clinical documents for exchange)^6^. As explained, the CDA includes the raw spirometric data in accordance with an HL7 (Health Level 7 International) standard.

**Figure 1S**


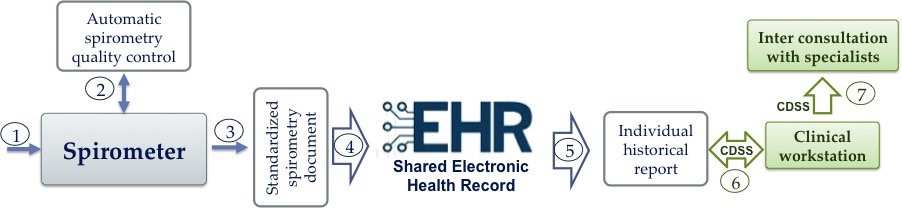


Figure 1S proposes logical workflow to allow sharing FS testing results (with improved quality assessment) irrespective of supplier and healthcare level.

**Figure 2S**


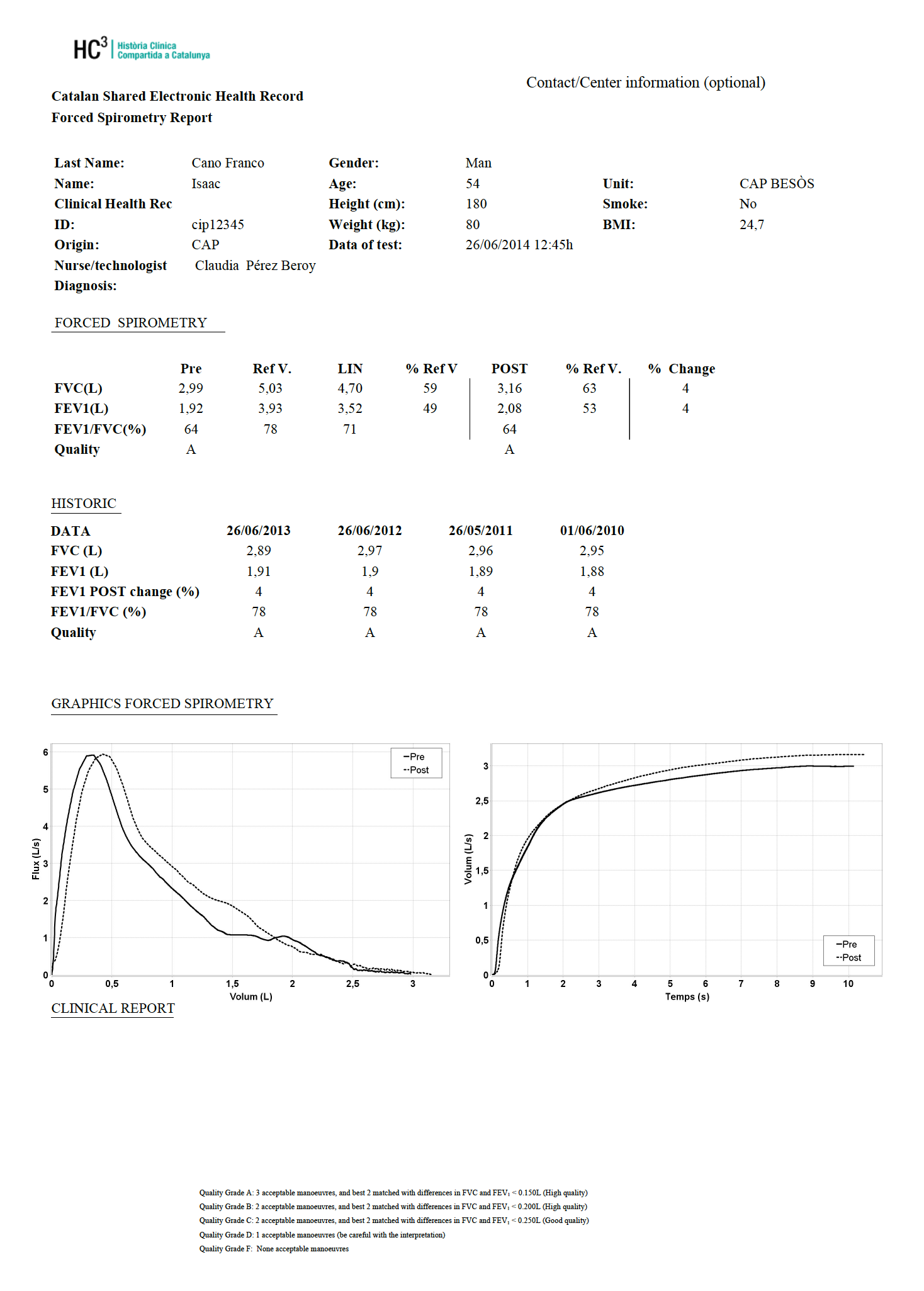


Figure 2S. Proposed individual historical report displaying the results of the last FS test and previous patient information on selected variables obtained from the shared electronic healthcare record at regional level (HC^3^)

**Section 2 – Forced spirometry report and software (Java) prototype for the creation of the document**

The **software (Java) prototype for the creation of the forced spirometry report with historic** of past principal spirometric variables can be downloaded via the following link:


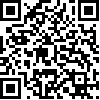


<http://bit.do/CDA2HPDF_ENG>

This prototype generates from an actual forced spirometry test (in principle, the most recent of the citizen), a PDF report including the historical values of FVC, FEV1, FEV1, FEV1/FVC change POST and quality taken from up to six previous forced spirometry test. All forced spirometry tests are assumed to be stored on a local disk and structured in accordance with the HL7 implementation guide for CDA R2 forced spirometry (included as part of this online supplementary material).

To test the prototype, an actual forced spirometry test (2273.xml) and four previous forced spirometry tests (22731.xml, 22732.xml, 22733.xml, 22734.xml) is included in this release.

The following file tree corresponds to the software prototype (once downloaded and extracted de content of the zip file):


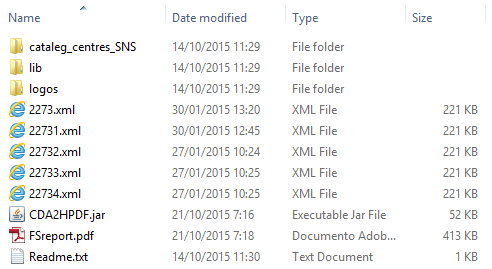


As explained in the *Readme.txt* text file, to test the prototype, the following command line can be executed in the command prompt (Windows operating systems):

java -jar CDA2HPDF.jar test

The test option generates a forced spirometry report *FSreport.pdf* from the actual forced spirometry (2273.xml) and the four previous forced spirometry (22731.xml, 22732.xml, 22733.xml, 22734.xml) example tests.

The general format of the arguments is the following:

java -jar CDA2HPDF.jar actualFS.xml previousFS_1.xml previousFS_N.xml
 nameFSreport.pdf

**Section 3. Assessment of the deployment of Forced Spirometry program (Table 1S) and long-term evaluation of the program beyond the deployment phase (Table 2S)**

**Table 1S.** Case Report Form for assessment of the FS program during 2016

| **MAST Domain** | **Indicators** | **Tool** | **Population** | **Time points assessment** |
| --- | --- | --- | --- | --- |
| Clinical Safety | Technical dysfunction with clinical impact | Questionnaire | All patients | Event |
| Clinical Effectiveness | Ascertainment of COPD or Asthma diagnosis (% diagnosis supported by FS) | Shared EHR tracking | All patients | Each PDSA cycle |
| Professionals perspectives | Overall satisfaction & usability | Specific questionnaire | All professionals | Each PDSA cycle |
| Economic aspects | Incremental cost-effectiveness ratio (ICER)^1^ | NA | 12-months | |
|  | Impact of the intervention on working life |  |  |  |
|  | Influence of the service to the way care is being provided |  |  |  |
|  | Change of liability due to the characteristics of the service |  |  |  |
| Organizational aspects | Staff allocation to tasks | Focus group & interviews | All professionals | Each PDSA cycle |
|  | Staff attitudes towards new services |  |  |  |
|  | Cooperation in tasks and work |  |  |  |
|  | Managerial issues |  |  |  |
| Socio-cultural, ethical & legal issues | Influence of the service to the way care is being provided | Focus group | All professionals | Each PDSA cycle |
|  | Change of liability due to the characteristics of the service |  |  |  |
| Transferability | Geographical | Assessment of four core items (Figure 1) | NA | Baseline |

**Table 2S.** Key indicators for long-term monitoring of adoption of the Forced Spirometry program beyond the regional deployment

| **Dimensions** | **Indicator** | **Tool** | **Variable, units** |
| --- | --- | --- | --- |
| **Impact on subjects**  *(individual health indicators)* | - Ascertainment of COPD or Asthma diagnosis | - FS testing | - Performance of FS testing (Yes/No) |
| **Maturity of the process implementation**  *(process indicators)* | - Professionals actively using the FS program | - Shared EHR and Provider EHR | - Number and percentage of potential professionals included in the service |
|  | - Primary Care Units actively using the FS program | - Shared EHR and Provider EHR | - Number and percentage of organizational units providing the service |
|  | - Distribution of maturity scores of the FS program (graded 1 to 3) | - Shared EHR and Provider EHR | - Percentage of organizational units in each of the three maturity scores (1-3) |
| **Impact on society**  *(healthcare outcome & value generation indicators)* | - Quality of FS testing | - Shared EHR and Provider EHR | - Distribution of quality scores |
|  | - Duplication of FS testing | - Shared EHR and Provider EHR | - Number of FS tests within a given internal |
|  | - Ascertainment of COPD and Asthma diagnosis at health system level | - Shared EHR and Provider EHR | - Percentage of COPD / asthma cases without FS test |

**Section 4. Standardized FS testing information. Implementation guide using a Clinical Document Architecture (CDA)**

CDA R2 implementation guide of the Forced Spirometry test


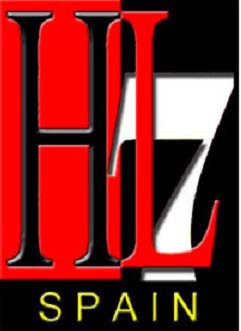


**Version 3.0.1 21/10/2012**

**English translation version 1.0 06/09/2015**

**Track Changes**

| **Version** | **Description** | **Author** | **Date** |
| --- | --- | --- | --- |
| 3.01 | Translation to English of version 3.01 | I. Cano | 06/09/15 |
| 3.01 | Formatted according to the guides HL7 template | A. Sáez | 21/10/12 |
| 3.00 | Final version of the Guide by the group constituted by the Technical Committee of HL7 SPAIN | A.Martínez,  M.Domingo, I.Petrus | 25/05/12 |
| 2.04 | Revision of the appendices by the group constituted by the Technical Committee of HL7 SPAIN | A.Martínez,  M.Domingo, I.Petrus | 04/04/12 |
| 2.03 | Review of encoded entries by the group constituted by the Technical Committee of HL7 SPAIN | A.Martínez,  M.Domingo, I.Petrus | 01/03/12 |
| 2.02 | Review of the document main body by the group constituted by the Technical Committee of HL7 SPAIN | A.Martínez,  M.Domingo, I.Petrus | 12/01/12 |
| 2.01 | Review of the document header by the group constituted by the Technical Committee of HL7 SPAIN | A.Martínez,  M.Domingo, I.Petrus | 25/11/11 |
| 2.00 | Review of the document introduction by the group constituted by the Technical Committee of HL7 SPAIN | A.Martínez,  M.Domingo, I.Petrus | 02/11/11 |
| 1.52 | Final guide format | M.Domingo | 24/10/11 |
| 1.51 | New translation of the final part | M.Lizana | 17/10/11 |
| 1.50 | New translation of the initial part of the Guide | I.Petrus | 17/10/11 |
| 1.49 | Revision of the translation done | A.Martínez | 13/10/11 |
| 1.48 | Translation of the final part of the Guide | M.Lizana | 10/10/11 |
| 1.47 | Translation of the initial part of the Guide | I.Petrus | 10/10/11 |
| 1.46 | Add element to display graphs on outside page to CDA | M.Lizana | 02/06/11 |
| 1.45 | General revision (Templates, OIDS’s, etc.) | M.Domingo | 20/04/11 |
| 1.44 | Add *organizers* in graphs section | M.Domingo | 16/04/11 |
| 1.43 | Graphs inserted B64 | M.Lizana | 24/03/11 |
| 1.42 | Application of the Gencat template | M.Domingo | 08/03/11 |
| 1.41 | Units update and RH-17 | M.Domingo | 08/03/11 |
| 1.40 | Update of the fields | M.Domingo | 04/02/11 |
| 1.30 | Review | R.Liñán | 31/01/11 |
| 1.01 | Complete version | M.Domingo | 27/01/11 |
| 1.00 | Format of the Standards and Interoperability office | M.Domingo | 23/11/10 |
| 0.99 | Fields modified | M. Lizana | 13/09/10 |
| 0.70 | OIDs | D. Kaminker | 07/04/10 |
| 0.48 | Coded entries SNOMED / Templates | D. Kaminker | 05/04/10 |
| 0.19 | Sections | D. Kaminker | 08/03/10 |
| 0.2 | Initial version | D. Kaminker | 08/03/10 |

**Table of Contents**

[1. Introduction 5](#__RefHeading___Toc421700758)

[**1.1.** **Object** 5](#__RefHeading___Toc421700759)

[**1.2.** **Scope** 5](#__RefHeading___Toc421700760)

[**1.3.** **Audience** 5](#__RefHeading___Toc421700761)

[**1.4.** **Methodology** 5](#__RefHeading___Toc421700762)

[**1.5.** **Conventions used in this Guide** 5](#__RefHeading___Toc421700763)

[**1.6.** **XPATH Notation** 6](#__RefHeading___Toc421700764)

[**1.7.** **Keywords** 6](#__RefHeading___Toc421700765)

[**1.8.** **XML examples** 6](#__RefHeading___Toc421700766)

[**1.9.** **Artifacts of the Implementation Guide** 6](#__RefHeading___Toc421700767)

[**1.10. Identification of documents in accordance with this guide** 7](#__RefHeading___Toc421700768)

[2. Composition of the HL7 Sub-Committee on spirometry 8](#__RefHeading___Toc421700770)

[3. Detail of the restrictions in this guide 9](#__RefHeading___Toc421700771)

[3.1. RH – Header restrictions 9](#__RefHeading___Toc421700772)

[3.1.1. Root element 9](#__RefHeading___Toc421700773)

[3.1.2.ClinicalDocument/typeId 9](#__RefHeading___Toc421700774)

[3.1.3.ClinicalDocument/templateId 9](#__RefHeading___Toc421700775)

[3.1.4.ClinicalDocument/id 10](#__RefHeading___Toc421700776)

[3.1.5.ClinicalDocument/code 10](#__RefHeading___Toc421700777)

[3.1.6.ClinicalDocument/title 10](#__RefHeading___Toc421700778)

[3.1.7.ClinicalDocument/effectiveTime 11](#__RefHeading___Toc421700779)

[3.1.8.ClinicalDocument/confidentialityCode 11](#__RefHeading___Toc421700780)

[3.1.9.ClinicalDocument/languageCode 11](#__RefHeading___Toc421700781)

[3.1.10.ClinicalDocument/setId and ClinicalDocument/versionNumber 12](#__RefHeading___Toc421700782)

[3.1.11.ClinicalDocument/recordTarget/patientRole 12](#__RefHeading___Toc421700783)

[3.1.12. ClinicalDocument/author/[1]Spirometer 13](#__RefHeading___Toc421700784)

[3.1.13. ClinicalDocument/author/[2]Clinican 16](#__RefHeading___Toc421700785)

[3.1.14. ClinicalDocument/custodian 19](#__RefHeading___Toc421700786)

[3.1.15.ClinicalDocument/informationRecipient 20](#__RefHeading___Toc421700787)

[3.1.16.ClinicalDocument/infullfillmentOf 23](#__RefHeading___Toc421700788)

[3.1.17.ClinicalDocument/documentationOf 23](#__RefHeading___Toc421700789)

[3.2. RB – RESTRICTIONS FOR THE BODY 25](#__RefHeading___Toc421700790)

[3.2.1. RB-S001 – PATIENT DATA SECTION 26](#__RefHeading___Toc421700791)

[3.2.2. RB-S002 – SECTION FOR THE CONTEXT OF THE FS TEST 27](#__RefHeading___Toc421700792)

[3.2.3. RB-S003 – SECTION FOR THE RESULTS OF THE FS TEST 29](#__RefHeading___Toc421700793)

[3.2.4. RB-S004 – SECTION FOR THE RESULTS BY MANOEUVRE 31](#__RefHeading___Toc421700794)

[3.2.5. RB-S005 – SECTION FOR THE FS SIGNALS 35](#__RefHeading___Toc421700795)

[3.2.6. RB-S006 – SECTION FOR COMMENTS 38](#__RefHeading___Toc421700796)

[3.2.7. RB-S007 – LINK TO THE GRAPHS 39](#__RefHeading___Toc421700797)

[3.3. RC – RESTRICTION FOR THE CODED ENTRIES 40](#__RefHeading___Toc421700798)

[3.3.1. RC-01 – PATIENT DATA SECTION 40](#__RefHeading___Toc421700799)

[3.3.2. RC-02 – SECTION FOR THE CONTEXT OF THE FS TEST 41](#__RefHeading___Toc421700800)

[3.3.3. RC-03 – SECTION FOR THE RESULTS OF THE FS TEST 42](#__RefHeading___Toc421700801)

[3.3.4. RC-04 – SECTION FOR THE RESULTS BY MANOEUVRE 43](#__RefHeading___Toc421700802)

[3.3.5. RC-05 – SECTION FOR THE FS SIGNALS 47](#__RefHeading___Toc421700803)

[3.3.6. RC-06 – SECTION FOR COMMENTS 48](#__RefHeading___Toc421700804)

[4. APPENDIX 49](#__RefHeading___Toc421700805)

[4.1. APPENDIX I – DETAIL OF CONSIDERED OIDs 49](#__RefHeading___Toc421700806)

[4.2. APPENDIX II – LOCAL VOCABULARIES 50](#__RefHeading___Toc421700807)

[4.3. APPENDIX III – REFERENCES TO INTERNATIONAL VOCABULARIES 54](#__RefHeading___Toc421700808)

[4.4. APPENDIX IV – TEMPLATES FOR CODED ENTRIES 55](#__RefHeading___Toc421700809)

[4.4.1. MEASUREMENTS TEMPLATES 55](#__RefHeading___Toc421700810)

[4.4.2. TEMPLATES FOR ANSWERS 58](#__RefHeading___Toc421700811)

# Introduction

## Object

The purpose of this document is to describe the restrictions to apply in the CDA R2 documents (header, body and encoded entries) that must be transmitted for the Forced Spirometry (FS) tests.

## Scope

This document describes the restrictions to be applied to the CDA R2 documents for FS reports generated in the healthcare network. FS is a physiological test that assesses the capacity of the lungs to inhale or exhale air. This capability is determined by measuring the volume per unit of time and flow.

## Audience

This document is intended for the development teams responsible for reporting FS tests.

## Methodology

The methodology is based on the revised guides and similar templates for CDA R2 at local and international level.

## Conventions used in this Guide

The standard on which this guide is based is HL7 Clinical Document Architecture, Release 2.0. As defined in this document, this is a guide for implementation that restricts the tags and vocabularies. Conformity requirements appear in this guide in the following format, being numbered consecutively.

- **RH-n:** This is an example of requirements in accordance to the **header of the document**.
- **RB-n:** This is an example of the requirement of conformity to the **body of the document**.
- **RC-n:** this is an example of requirement of conformity for actionable **coded entries**.

## XPATH Notation

This guide uses XPATH notation for compliance predicates and in any place were XML attributes are referenced or identified. The implicit context is the root of the document. It is meant to provide a familiar mechanism for developers to identify parts of an XML document were restrictions could be applied.

## Keywords

The following are some clarifications on the meaning of the use of the verbs "must" and "can" in the statement of requirements, aligned with the features "mandatory" and "optional" of the data model:

- **Must**: indicates that this rule is mandatory.
- **Can:** indicates that if present is accepted as valid, but if it is not present there is no reason to reject the document. It is therefore optional.

**Note:** In some cases, the data set is optional in the model, but if you include it, some or all of its constituent elements are mandatory.

In case an element should include a fixed or invariant value, it must be expressed as *[fixed value]*.

## XML examples

The XML examples that appear in this document are included with a fixed-size font for easy reading. The entire contents of the fragment is not provided - if you want to see the full content of the example, please see attached examples.

*<ClinicalDocument xmlns:xsi="*[*http://www.w3.org/2001/XMLSchema-instance*](http://www.w3.org/2001/XMLSchema-instance)*" xsi:schemaLocation="urn:hl7-org:v3 CDA.xsd" xmlns:voc="urn:hl7-org:v3/voc"*

*xmlns="urn:hl7-org:v3">*

*:*

*</ClinicalDocument>*

## Artifacts of the Implementation Guide

| **Fichero** | **Description** |
| --- | --- |
| CDA_Espirometria _V3.0.2.doc | This Guide for implementation (in English) |
| CdaCompleto.xml | Example of a CDA R2 compliance document. Valid for the baseline and the bronchodilator tests (Full Version). |
| ESP_VIEW.xsl | Style sheets to display the examples provided in XML format. |

## 1.10. Identification of documents in accordance with this guide

CDA provides a mechanism to indicate the reference to an implementation guide with an identifier assigned by an organization enabled to do so.

The table that follows shows the identifier associated with this guide.

| **Extension** | **OID (root)** |
| --- | --- |
| T00 | 2.16.840.1.113883.2.19.60.2 |

The following example shows how to indicate formally that an instance of a CDA document is intended to be compliance to this implementation guide:

*<ClinicalDocument xmlns:xsi="*[*http://www.w3.org/2001/XMLSchema-instance*](http://www.w3.org/2001/XMLSchema-instance)*" xsi:schemaLocation="urn:hl7-org:v3 CDA.xsd" xmlns:voc="urn:hl7-org:v3/voc"*

*xmlns="urn:hl7-org:v3">*

*<typeId extension='POCD_HD000040' root='2.16.840.1.113883.1.3'/>*

***<templateId extension='T00' root='2.16.840.1.113883.2.19.60.2'/>***

*:*

*</ClinicalDocument>*

# Composition of the HL7 Sub-Committee on spirometry

| **Member** | **Organization** |
| --- | --- |
| Manel Domingo | TecnoCampos Matró-Maresme  Centre de Compètencies d’Integració TicSalut  Departament de Salut Generalitat de Calalunya |
| Alicia Martínez | Hospital Universitario Virgen del Rocío  Servicio Andaluz de Salud |
| Iván Petrus | iBit Salut  Centre Competències Integració Illes Balears  Innovació tencológica |

# Detail of the restrictions in this guide

## RH – Header restrictions

### Root element

The root of all documents to which this guide applies should be ClinicalDocument in the namespace urn:hl7-org.v3.

*<ClinicalDocument xmlns:xsi="*[*http://www.w3.org/2001/XMLSchema-instance*](http://www.w3.org/2001/XMLSchema-instance)*" xsi:schemaLocation="urn:hl7-org:v3 CDA.xsd" xmlns:voc="urn:hl7-org:v3/voc"*

*xmlns="urn:hl7-org:v3">*

*:*

*</ClinicalDocument>*

### 3.1.2.ClinicalDocument/typeId

##### [Fixed Value]

This element **must** be present. Identifies the underlying model of the document.

| **Restriction** | **Description** |
| --- | --- |
| ***RH-01*** | The element ClinicalDocument/typeId **must** be present. The element ClinicalDocument/typeId **must** contain the value:  @root=’2.16.840.1.113883.1.3’  @extension=’POCD_HD000040’ |

***<typeId extension='POCD_HD000040' root='2.16.840.1.113883.1.3'/>***

### 3.1.3.ClinicalDocument/templateId

##### [Fixed Value]

This element identifies the template that represents this implementation guide.

| **Restriction** | **Description** |
| --- | --- |
| ***RH-02*** | The element ClinicalDocument/templateId **must** be present with the value: @root='2.16.840.1.113883.2.19.60.2**'** and @extension=**'T00'** |

***<templateId extension='T00' root='2.16.840.1.113883.2.19.60.2'/>***

### 3.1.4.ClinicalDocument/id

This element must contain the unique identifier (ID) of the document in the system on which it was issued, expressed as @root = OID of the document identification generator of the issuing system, @extension = 'document identifier'. @extension must be unique within @root.

| **Restriction** | **Description** |
| --- | --- |
| ***RH-03*** | The ClinicalDocument/id/@root **must** be a registered and correct OID. @extension must be the document identifier.  More information on how to apply for an OID in Appendix I, paragraph "OID assignment for new producers or institutions willing to use the Guide”. |

### 3.1.5.ClinicalDocument/code

##### [Fixed Value]

| **Restriction** | **Description** |
| --- | --- |
| ***RH-04*** | ClinicalDocument/@code **must** be the value INF_ESP of the set of concepts defined locally in this guide, representing the document type: spirometry report.  ClinicalDocument/@codeSystem **must** be the value 2.16.840.1.113883.2.19.60.2.5. |

***<code code='INF_ESP' codeSystem='*2. 16. 840. 1. 113883. 2. 19. 60. 2. 5*'/>***

### 3.1.6.ClinicalDocument/title

| **Restriction** | **Description** |
| --- | --- |
| ***RH-05*** | The element title of the document **must** be present and contain the local title. |

***<title>Spirometry Report </title>***

### 3.1.7.ClinicalDocument/effectiveTime

This date references the moment in which the document or FS report was generated. It can be after the date of the FS test.

| **Restriction** | **Description** |
| --- | --- |
| ***RH-06*** | The element ClinicalDocument/effectiveTime of the document **must** be present and must have precision of seconds and the expression of the time zone. |

***<effectiveTime value='20100220100210+0000'/>***

### 3.1.8.ClinicalDocument/confidentialityCode

##### [Fixed Value]

| **Restriction** | **Description** |
| --- | --- |
| ***RH-07*** | The element ClinicalDocument/confidentialityCode **must** be present. The value of the @confidentialityCode attribute **must** be N. The value of the @codeSystem attribute **must** be '2.16.840.1.113883.5.25'. |

***<confidentialityCode code='N' codeSystem='2.16.840.1.113883.5.25'/>***

### 3.1.9.ClinicalDocument/languageCode

M**ust** be present and represent the language of document.

| **Restriction** | **Description** |
| --- | --- |
| ***RH-08*** | The element languageCode **must** be present and with the attribute @code with format “xx-ES”, where xx must be one of the values listed in the ISO-639-1 table. |

***<languageCode code='es-ES'/>***

### 3.1.10.ClinicalDocument/setId and ClinicalDocument/versionNumber

May be present in case that the FS document issuing system supports the generation of different report versions for the same FS. In this case, the issuing system must generate both elements in all documents.

| **Restriction** | **Description** |
| --- | --- |
| ***RH-09*** | ClinicalDocument/setId and ClinicalDocument/versionNumber **must** be present or not (both). |

| **Restriction** | **Description** |
| --- | --- |
| ***RH-10*** | The element versionNumber/@value **must** increase one by one in case of generating a new document version. The element setId **must** be equal to the original document. |

### 3.1.11.ClinicalDocument/recordTarget/patientRole

Identified the patient to whom the FS was performed.

| **Restriction** | **Description** | | |
| --- | --- | --- | --- |
| ***RH-11*** | **Must** be only one element ClinicalDocument/recordTarget/patientRole |  |  |

| **Restriction** | **Description** |
| --- | --- |
| ***RH-12-A*** | The inclusion of the patient identification is required. The attribute @extension of the element patientRole/id **must** be the personal identification code (CIP) of the patient. The value @root **must** be 2.16.724.4.41 (OID for the personal identification code). |

| **Restriction** | **Description** |
| --- | --- |
| ***RH-12-B*** | **Can** be other elements patientRole/id optionally. Each element id **must** include in the @root attribute the OID that identifies the type of id used (standard or from a local vocabulary). The attribute @extension **must** be the id of the patient. |

| **Restriction** | **Description** |
| --- | --- |
| ***RH-13*** | At least one element patient/name/given **must** be present and contain the name of the patient. If needed, patient/name **can** have another element given for a second patient name. |

| **Restriction** | **Description** |
| --- | --- |
| ***RH-14*** | At least one element patient/name/family **must** be present and contain the surname of the patient. If needed, patient/name **can** have another element family for a second patient surname. |

| **Restriction** | **Description** |
| --- | --- |
| ***RH-15*** | The element patient/administrativeGenderCode **can** be present and must belong to the HL7 vocabulary. |

| **Restriction** | **Description** |
| --- | --- |
| ***RH-16*** | The element patient/birthTime **must** be present and with days precision. |

| **Restriction** | **Description** |
| --- | --- |
| ***RH-17*** | The element patientRole/telecom **must** be present and must contain patient telecommunication information (e.g., telephone or email). |

| **Restriction** | **Description** |
| --- | --- |
| ***RH-18*** | The element patientRole/addr **can** be present and must contain the postal address of the patient. |

### 3.1.12. ClinicalDocument/author/[1]Spirometer

With respect to the author, for CDA R2 at least one instance is mandatory. In the case of this Guide, the only mandatory author is the spirometer, because both the technician and the clinician are optional.

| **Restriction** | **Description** |
| --- | --- |
| ***RH-19*** | The element author **must** be present. |

| **Restriction** | **Description** |
| --- | --- |
| ***RH-20*** | The element author/time **must** be present. Indicates the date of the FS test performance, even if the technician or the doctor may have loaded more information afterwards. Precision: seconds. |

| **Restriction** | **Description** |
| --- | --- |
| ***RH-21*** | The element assignedAuthor/id **must** be present and must contain the attributes @root and @extension that identifies unambiguously the spirometer. |

| **Restriction** | **Description** |
| --- | --- |
| ***RH-22*** | The element assignedAuthor **must** contain an element assignedAuthoringDevice/code, indicating the type of device. Fixed value: code=”303501006” codeSystem=”2.16.840.1.113883.6.96”  codeSystemName=”SNOMED CT” displayName=”Spirometer” |

| **Restriction** | **Description** |
| --- | --- |
| ***RH-23*** | The element assignedAuthor **must** contain an element assignedAuthoringDevice/manufacturerModelName, indicating the model of the device. |

| **Restriction** | **Description** |
| --- | --- |
| ***RH-24*** | The element assignedAuthor **must** contain an element assignedAuthoringDevice/softwareName, indicating the name and version of the software. |

| **Restriction** | **Description** |
| --- | --- |
| ***RH-25*** | The element assignedAuthor/representedOrganization **must** be present. It represents the organization in which the FS has been performed. |

| **Restriction** | **Description** |
| --- | --- |
| ***RH-26-A*** | The element assignedAuthor/representedOrganization/id **must** be present, with attributes:  For hospitals @root=2.16.724.4.21.5.1 and @extension=one of the codes assigned in the national catalogue of hospitals published in the website of the Ministry.  For primary care centres @root=2.16.724.4.21.5.2 and @extension= One of the codes assigned in the catalogue of primary care centres from the NHS published in the website of the Ministry. |

| **Restriction** | **Description** |
| --- | --- |
| ***RH-26-B*** | Optionally, other assignedAuthor/representedOrganization/id elements **can** be present. Each element id **must** include in the @root attribute the OID that identifies the type of id used (standard or from the local vocabulary). The attribute @extension must be the id of the organisation. |

| **Restriction** | **Description** |
| --- | --- |
| ***RH-27*** | The element assignedAuthor/representedOrganization/name **can** be present with the name of the Organisation.  estar presente con el nombre de la organización. |

| **Restriction** | **Description** | | |
| --- | --- | --- | --- |
| ***RH-28*** | The | element | assignedAuthor/representedOrganization/telecom **can** be |

present and must contain the telecommunication contact information (e.g., telephone/email) of the Organisation.

| **Restriction** | **Description** |
| --- | --- |
| ***RH-29*** | The element assignedAuthor/representedOrganization/addr **can** be present and must contain the postal address of the organisation. |

### 3.1.13. ClinicalDocument/author/[2]Clinican

Optional element identifying the clinician validating the FS test.

| **Restriction** | **Description** |
| --- | --- |
| ***RH-30*** | A second element author **can** be present, representing the clinician that validates the FS test. In this case, its internal elements (ID, surname, name, validation date) are mandatory. |

| **Restriction** | **Description** |
| --- | --- |
| ***RH-31*** | The element author/time **must** be present. It indicates the date of the test validation. Precision: seconds. |

| **Restriction** | **Description** |
| --- | --- |
| ***RH-32-A*** | The element assignedAuthor/id **must** be present and must contain the attributes @root and @extension that identify unambiguously the clinician that validates the FS test.  que valida (nro. de colegiado) |

| **Restriction** | **Description** |
| --- | --- |
| ***RH-32-B*** | Optionally, other assignedAuthor/id elements **can** be present. Each id element must include in the @root attribute the OID that identifies the type of id used (standard or from the local vocabulary). The attribute @extension must be the id of the clinician (clinician number). |

| **Restriction** | **Description** |
| --- | --- |
| ***RH-33*** | The element assignedAuthor/telecom **can** be present and must contain the telecommunication contact information (e.g., telephone/email) of the clinician. |

| **Restriction** | **Description** |
| --- | --- |
| ***RH-34*** | The element assignedAuthor/code **can** be present and must contain the code of the clinicians’ role. |

| **Restriction** | **Description** |
| --- | --- |
| ***RH-35*** | The element assignedAuthor **must** contain at least one element assignedPerson/name/family, containing the surname of the clinician that validated the FS test. The element assignedAuthor **can** contain more than one element assignedPerson/name/family, containing additional surnames of the clinician that validates the FS test. |

| **Restriction** | **Description** |
| --- | --- |
| ***RH-36*** | The element assignedAuthor **must** contain at least one element assignedPerson/name/given, containing the name of the clinician that validated the FS test. The element assignedAuthor **can** contain more than one element assignedPerson/name/given, containing additional names of the clinician that validates the FS test. |

| **Restriction** | **Description** |
| --- | --- |
| ***RH-37-A*** | The element assignedAuthor/representedOrganization/id **must** be present, with attributes:  For hospitals @root=2.16.724.4.21.5.1 and @extension=one of the codes assigned in the national catalogue of hospitals published in the website of the Ministry.  For primary care centres @root=2.16.724.4.21.5.2 and @extension= One of the codes assigned in the catalogue of primary care centres from the NHS published in the website of the Ministry. |

| **Restriction** | **Description** |
| --- | --- |
| ***RH-37-B*** | Optionally, other assignedAuthor/representedOrganization/id elements **can** be present. Each element id **must** include in the @root attribute the OID that identifies the type of id used (standard or from the local vocabulary). The attribute @extension must be the id of the organisation. |

| **Restriction** | **Description** |
| --- | --- |
| ***RH-38*** | The element assignedAuthor/representedOrganization/name **can** be present. Represents the service for which the clinician that validates the FS test works – can be equal to the service were the FS test is performed. |

| **Restriction** | **Description** |
| --- | --- |
| ***RH-39*** | The element assignedAuthor/representedOrganization/telecom **can** be present and must contain the telecommunication contact information (e.g., telephone/email) of the organization. |

| **Restriction** | **Description** |
| --- | --- |
| ***RH-40*** | The element assignedAuthor/representedOrganization/addr **can** be present and must contain the postal address of the organisation. |

### 3.1.14. ClinicalDocument/custodian

Represents the Organisation that generates and preserves the original documents.

| **Restriction** | **Description** |
| --- | --- |
| ***RH-41*** | The element custodian **must** be present. |

| **Restriction** | **Description** |
| --- | --- |
| ***RH-42-A*** | The element custodian **must** contain one element  assignedCustodian/representedCustodianOrganization/id that contains the identifier of the Organisation responsible for the storage of the FS report. |

| **Restriction** | **Description** |
| --- | --- |
| ***RH-42-B*** | Optionally, the element custodian **can** contain other  assignedCustodian/representedCustodianOrganization/id elements. Each id element must include in the @root attribute the OID that identifies the type of id used (standard or from the local vocabulary). The attribute @extension must be the id of the organisation. |

| **Restriction** | **Description** |
| --- | --- |
| ***RH-43*** | The element assignedCustodian/representedCustodianOrganization/name in the element custodian **can** be present, representing the name of the Organisation. |

| **Restriction** | **Description** |
| --- | --- |
| ***RH-44*** | The element assignedCustodian/representedCustodianOrganization/telecom in the element custodian **can** be present and must contain the telecommunication contact information of the Organisation. |

| **Restriction** | **Description** |
| --- | --- |
| ***RH-45*** | The element assignedCustodian/representedCustodianOrganization/addr **can** be present and must contain the postal address of the organisation. |

### 3.1.15.ClinicalDocument/informationRecipient

Indicates the Organisation and professional that requested the FS test and must contain a copy of the FS report – this element is optional.

| **Restriction** | **Description** |
| --- | --- |
| ***RH-46*** | One element clinicalDocument/informationRecipient **can** be present, representing the clinician, organisation and service that requests the FS test. If present, its internal elements (ID, surname, name, organisation and service) are mandatory. |

| **Restriction** | **Description** |
| --- | --- |
| ***RH-47-A*** | The element intendedRecipient/id **must** be present and must contain the attributes @root and @extension that identify unambiguously the clinician that requests the FS test (clinician number). |

| **Restriction** | **Description** |
| --- | --- |
| ***RH-47-B*** | Optionally, other intendedRecipient/id elements **can** be present. Each id element must include in the @root attribute the OID that identifies the type of id used (standard or from the local vocabulary). The attribute @extension must be the id of the clinician. |

| **Restriction** | **Description** |
| --- | --- |
| ***RH-48*** | The element intendedRecipient/informationRecipient/name **must** contain at least one element family, indicating the surname of the clinician that requests the FS test.  The element intendedRecipient/informationRecipient/name **can** contain more than one element family, indicating additional surnames of the clinician that requests the FS test. |

| **Restriction** | **Description** |
| --- | --- |
| ***RH-49*** | The element intendedRecipient/informationRecipient/name **must** contain at least one element given, indicating the name of the clinician that requests the FS test.  The element intendedRecipient/informationRecipient/name **can** contain more than one element given, indicating additional names of the clinician that requests the FS test. |

| **Restriction** | **Description** |
| --- | --- |
| ***RH-50*** | The element intendedRecipient/receivedOrganization **can** be present, indicating the name of the service that requests the FS test. If present, intendedRecipient/receivedOrganization/id **must** be present. |

| **Restriction** | **Description** |
| --- | --- |
| ***RH-51-A*** | The element intendedRecipient/receivedOrganization/id **must** be present, with attributes: |

For hospitals @root=2.16.724.4.21.5.1 and @extension=one of the codes

assigned in the national catalogue of hospitals published in the website of the Ministry.

For primary care centres @root=2.16.724.4.21.5.2 and @extension= One of the codes assigned in the catalogue of primary care centres from the NHS published in the website of the Ministry.

| **Restriction** | **Description** |
| --- | --- |
| ***RH-51-B*** | Optionally, other intendedRecipient/receivedOrganization/id elements **can** be present. Each element id **must** include in the @root attribute the OID that identifies the type of id used (standard or from the local vocabulary). The attribute @extension must be the id of the organisation. |

| **Restriction** | **Description** |
| --- | --- |
| ***RH-52*** | The element intendedRecipient/receivedOrganization/name **can** be present, indicating the name of the service that requests the FS test. |

| **Restriction** | **Description** |
| --- | --- |
| ***RH-53*** | The element intendedRecipient/receivedOrganization/telecom **can** be present and must contain the telecommunication contact information (e.g., telephone/email) of the organization. |

| **Restriction** | **Description** |
| --- | --- |
| ***RH-54*** | The element intendedRecipient/representedOrganization/addr **can** be present and must contain the postal address of the organisation. |

| **Restriction** | **Description** |
| --- | --- |
| ***RH-55-A*** | The element  intendedRecipient/receivedOrganization/asOrganizationPartOf/id  **can** be present, indicating the identification of the organisation that requests the FS test. |
| **Restriction** | **Description** |
| ***RH-55-B*** | Optionally, other  intendedRecipient/receivedOrganization/asOrganizationPartOf/id elements **can** be present. Each element id **must** include in the @root attribute the OID that identifies the type of id used (standard or from the local vocabulary). The attribute @extension must be the id of the organisation. |

| **Restriction** | **Description** |
| --- | --- |
| ***RH-56*** | The elementintendedRecipient/receivedOrganization/asOrganizationPartOf/ wholeOrganization/name **can** be present, indicating the name of the organisation that requests the FS test. |

### 3.1.16.ClinicalDocument/infullfillmentOf

Indicates the number of the request to perform the FS test. This element is **mandatory**. It relates the FS test data with the rest of the clinical information of the patient (care process). The spirometer receives this information from the information system of the organisation.

| **Restriction** | **Description** |
| --- | --- |
| ***RH-57*** | The element ClinicalDocument/infullfillmentOf/order/id **must** be present indicating the number of the request to perform the FS test. The attribute @root contains the identifier of the numbering system and the attribute @extension the number of the request. |

### 3.1.17.ClinicalDocument/documentationOf

Contains the following information about the FS test performed: date stamp, time stamp, type of test, identifier of the test, technician performing the test.

| **Restriction** | **Description** |
| --- | --- |
| ***RH-58*** | The element ClinicalDocument/documentationOf/serviceEvent **must** be present. |

| **Restriction** | **Description** |
| --- | --- |
| ***RH-59*** | The element ClinicalDocument/documentationOf/serviceEvent/id **must** be present indicating the number of the FS test, generated by the spirometer or the departmental system. The attribute @root contains the identification of the numbering system and the attribute @extension the number of the test. |

| **Restriction** | **Description** |
| --- | --- |
| ***RH-60*** | The element ClinicalDocument/documentationOf/serviceEvent/code **must** be present indicating the type of the FS test. The attribute @code must exist in the table 2.16.840.1.113883.2.19.60.2.4, the codeSystem must be 2.16.840.1.113883.2.19.60.2.4 and the attribute @displayName must be the description associated to the code. |

| **Restriction** | **Description** |
| --- | --- |
| ***RH-61*** | The element  ClinicalDocument/documentationOf/serviceEvent/effectiveTime **must** be present. It indicates the date when the FS test was performed, even if the technician or clinician have added other information to the FS test afterwards. Precision: seconds. |

| **Restriction** | **Description** |
| --- | --- |
| ***RH-62-A*** | The element ClinicalDocument/documentationOf/serviceEvent/ performer/assignedEntity/id **can** be present.  Contains the ID of the technician or nurse who performs the test. The @root and @extension attributes must identify unambiguously the technician (Nr. of employee at the Medical Centre). |

| **Restriction** | **Description** |
| --- | --- |
| ***RH-62-B*** | Optionally, other elements ClinicalDocument/documentationOf/ serviceEvent/performer/assignedEntity/id **can** be present. Each id element must include in the @root attribute the OID that identifies the type of id used (standard or from the local vocabulary). The attribute @extension must be the id of the technician. |

| **Restriction** | **Description** |
| --- | --- |
| ***RH-63*** | The element  ClinicalDocument/documentationOf/serviceEvent/performer/assign edEntity/name **must** contain at least one element family, indicating the surname of the technician or nurse performing the FS test.  The element  ClinicalDocument/documentationOf/serviceEvent/performer/assign edEntity/name **can** contain more than one element family, indicating additional surnames of the technician or nurse performing the FS test. |

| **Restriction** | **Description** |
| --- | --- |
| ***RH-64*** | The element  ClinicalDocument/documentationOf/serviceEvent/performer/assign edEntity/name **must** contain at least one element given, indicating the name of the technician or nurse performing the FS test.  The element  ClinicalDocument/documentationOf/serviceEvent/performer/assign edEntity/name **can** contain more than one element given, indicating additional names of the technician or nurse performing the FS test. |

## RB – RESTRICTIONS FOR THE BODY

The document **must** express its narrative text using the structured body of the CDA R2. This section defines the restrictions required for the narrative block.

| **Restriction** | **Description** | | |
| --- | --- | --- | --- |
| ***RB-01*** | The document **must** contain an element  /ClinicalDocument/component/structuredBody. |  |  |

The structured body must contain three mandatory sections for data and can contain four optional sections for comments.

| **Restriction** | **Code** | **Section title** | **Requirement** |
| --- | --- | --- | --- |
| ***RB-02*** | S001 | Patient Data | Mandatory |
| ***RB-03*** | S002 | Spirometer Data | Optional |
| ***RB-04*** | S003 | FS test results | Mandatory |
| ***RB-05*** | S004 | Results for each manoeuvre | Optional |
| ***RB-06*** | S005 | Graphs and values of the signals | Mandatory |
| ***RB-07*** | S006 | Comments or other observations | Optional |
| ***RB-08*** | S007 | Links to the graphs | Optional |

In case section S004 exits, the restriction RB-05 with code S004 **must** be done for the basal test. If in addition the bronchodilator test if performed, the section **must** be duplicated with the same Restriction RB-05, by adding the suffix "0.1" to each field. The same applies to section RB-06.

### RB-S001 – PATIENT DATA SECTION

| **Restriction** | **Description** |
| --- | --- |
| ***RB-S001-***  ***01*** | The document **must** contain at least one section for the patient contextual data. This section must be identified with the code 'S001' of the local codification system for the sections of this document. The document **must** contain an element  /ClinicalDocument/component/structuredBody/component/section  /code/@code='S001' and @codeSystem='2.16.840.1.113883.2.19.60.2.1'  This section **must** contain the patient contextual information in textual form (requirement RB-S001) and coded (requirement RC-01). |

| **Restriction** | **Description** |
| --- | --- |
| ***RB-S001- 02*** | The section on personal data **must** contain the title DATOS PERSONALES (Personal data in Spanish) '/ClinicalDocument/component/structuredBody/component/section [@code='S001']/title'. |

| **Restriction** | **Description** |
| --- | --- |
| ***RB-S001-***  ***03*** | The document **can** contain textually the patient PROFESIÓN (occupation in Spanish) in the section 'S001'. |

| **Restriction** | **Description** |
| --- | --- |
| ***RB-S001-***  ***04*** | The document **can** contain textually the MOTIVO (reason in Spanish) of the FS test in the section 'S001'. |

| **Restriction** | **Description** |
| --- | --- |
| ***RB-S001-***  ***05*** | The document **must** contain textually the PESO (weight in Spanish) in KG of the patient in the section 'S001'. |

| **Restriction** | **Description** |
| --- | --- |
| ***RB-S001-***  ***06*** | The document **must** contain textually the EDAD (age in Spanish) in AÑOS (years in Spanish) of the patient in the section 'S001'. |

| **Restriction** | **Description** |
| --- | --- |
| ***RB-S001- 07*** | The document **must** contain textually the TALLA (height in Spanish) in METROS (meters in Spanish) of the patient in the section 'S001'. |

| **Restriction** | **Description** |
| --- | --- |
| ***RB-S001-***  ***08*** | The document **can** contain textually the GRUPO ETNICO (ethnic group in Spanish) of the patient in the section 'S001'. |

| **Restriction** | **Description** |
| --- | --- |
| ***RB-S001- 09*** | The document **can** contain textually the information about if the patient is FUMADOR (smoker in Spanish) or NO. |

#### Example of visualisation for the data in section S001 (in Spanish)

**DATOS DEL PACIENTE**


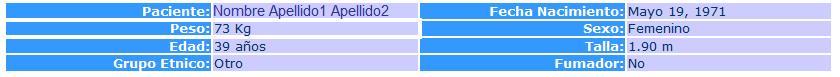


### RB-S002 – SECTION FOR THE CONTEXT OF THE FS TEST

| **Restriction** | **Description** |
| --- | --- |
| ***RB-S002-***  ***01*** | The document **can** contain at least one section for the contextual data of the FS test and the spirometer. This section must be identified with the code 'S002' of the local codification system for the sections of this document. The document **must** contain one element  /ClinicalDocument/component/structuredBody/component/section  /code/@code='S002' and @codeSystem='2.16.840.1.113883.2.19.60.2.1'  If this section exists, it **must** it must contain the contextual information of the spirometer, textually (requirement RB-S002) and coded (requirement RC-02). |

| **Restriction** | **Description** |
| --- | --- |
| ***RB-S002-***  ***02*** | The section on the FS test contextual data **must** contain the title DATOS DEL ESPIRÓMETRO (Spirometer data in Spanish)  '/ClinicalDocument/component/structuredBody/component/section [@code='S002']/title. |

| **Restriction** | **Description** |
| --- | --- |
| ***RB-S002- 03*** | The document **must** contain textually the type of TRANSDUCTOR in section 'S002'. The type of transducer must be listed in the vocabulary 2.16.840.1.113883.2.19.60.2.2. |

| **Restriction** | **Description** |
| --- | --- |
| ***RB-S002-***  ***04*** | The document **must** contain textually the calibration date, formatted as DD-MM-YYYY in section 'S002'. |

| **Restriction** | **Description** |
| --- | --- |
| ***RB-S002-***  ***05*** | The document **must** contain textually the calibration time, formatted as HH:MM:SS in section 'S002'. |

| **Restriction** | **Description** |
| --- | --- |
| ***RB-S002-***  ***06*** | The document **can** contain textually the TEMPERATURA AMBIENTE (Room temperature in Spanish) measured in ºC in section 'S002'. |

| **Restriction** | **Description** |
| --- | --- |
| ***RB-S002-***  ***07*** | The document **can** contain textually the PRESIÓN ATMOSFÉRICA (Atmospheric pressure in Spanish) measured in mm Hg in section 'S002'. |

| **Restriction** | **Description** | | | |
| --- | --- | --- | --- | --- |
| ***RB-S002-***  ***08*** | The document **can** contain textually the HUMEDAD RELATIVA AMBIENTE (Relative room humidity in Spanish) in section 'S002'. |  |  |  |

| **Restriction** | **Description** |
| --- | --- |
| ***RB-S002-***  ***09*** | The document **can** contain textually the name of the TABLA DE REFERENCIA (Reference table in Spanish) used to assess the FS test results. The values relating to the reference values used must be one of those listed in the table of this with OID 2.16.840.1.113883.2.19.60.2.7. |

#### Example visualisation of section S002 (in Spanish)

**DATOS DEL ESPIRÓMETRO**

**SPIROMETER DATA**

| **Tipo de Transductor**  **Transducer Type** | Fleisch |
| --- | --- |
|  | 10/12/2011 |
| **Fecha Calibración**  **Calibration data** |  |
|  |  |
| **Hora Calibración**  **Calibration time** | 07:30:00 |
| **Temperatura**  **Temperature** | 20 ºC |
|  | 760 mm Hg |
| **Presión Atmosférica**  **Atmospheric pressuer** |  |
|  |  |
| **Humedad**  **Humidity** | 60 % |
| **Tabla De Referencia**  **Utilizada**  **Reference Values** |  |
|  | ATS-ERS |
|  |  |

### RB-S003 – SECTION FOR THE RESULTS OF THE FS TEST

| **Restriction** | **Description** |
| --- | --- |
| ***RB-S003-***  ***01*** | The document **must** contain one section with the results of the FS test. It **must** be identified with the code 'S003' of the local codification system for the sections of this document. The document **must** contain one element  /ClinicalDocument/component/structuredBody/component/section  /code/@code='S003' and @codeSystem='2.16.840.1.113883.2.19.60.2.1'  This section **must** contain the general results of the study (not the specific results of each maneuver). This section **must** contain the information textually (requirements RB-S003) and coded (requirements RC-03).  Each field of this section **must** contain the values of the basal test. In addition, it **can** contain the bronchodilator test. In this case, it **must** contain the value of that test and the difference between the values of the two test (basal and bronchodilator). |

| **Restriction** | **Description** |
| --- | --- |
| ***RB-S003-***  ***02*** | The section about the results of the FS test **must** contain the title  RESULTADOS DEL ESTUDIO (Results of the study in Spanish)  '/ClinicalDocument/component/structuredBody/component/section [@code='S003']/title |

The section on the results of the FS test **must** contain textually each one of the parameters marked as **mandatory** on the "Requirement" column of the following table.

The section on the results of the FS test **can** contain textually each one of the parameters marked as **OPTIONAL** on the "Requirement" column of the following table.

| **Parameter** | **Description** | **Requirement** | **Unit** | **Restriction** |
| --- | --- | --- | --- | --- |
| Number of total manoeuvres | Number of total manoeuvres | Optional | {tot} | RB-S003-03 |
| Best FVC | Best FVC | Mandatory | L | RB-S003-04 |
| Expected FVC | Expected FVC according to the reference values used | Mandatory | L | *RB-S003-05* |
| % Best FVC /  Expected FVC | Ratio best FVC and expected FVC | Mandatory | % | *RB-S003-06* |
| Best FEV1 | Forced expired volume in the 1^st^ second second | Mandatory | L | *RB-S003-07* |
| Expected FEV1 | Expected FEV1C according to the reference tables used | Mandatory | L | *RB-S003-08* |
| % Best FEV1 /  Expected FEV1 | Ratio best FEV1 and expected FEV1 | Mandatory | % | RB-S003-09 |
| % Best FEV1 /  Best FVC | Ratio best FEV1 and best FVC | Mandatory | % | *RB-S003-10* |
| Quality control grade | Quality control grade | Optional |  | *RB-S003-11* |
| Best FEF25-75 | Best FEF25-75 | Optional | L/s | RB-S003-12 |
| Expected FEF25-75 | Expected FEF25-75 according to the reference values used | Optional | L/s | *RB-S003-13* |

| **Parameter** | **Description** | **Requirement** | **Unit** | **Restriction** |
| --- | --- | --- | --- | --- |
| % Best FEF25-75 /  Expected FEF25-75 | Ratio best FEF25-75 and expected FEF25-75 according to the reference values used | Optional | % | *RB-S003-14* |
| Best PEF | Best PEF | Optional | L/s | *RB-S003-15* |
| Expected PEF | Expected PEF according to the reference values used  las tablas de referencia utilizadas | Optional | L/s | *RB-S003-16* |
| % Best PEF /  Expected PEF | Ratio PEF and expected PEF | Optional | % | RB-S003-17 |
| Date and time | Date and time of the FS test execution | Optional | YYYYM  MDDHH MM | RB-S003-18 |

**Example visualisation of section S003 (in Spanish)**

**RESULTADOS DEL ESTUDIO**

**STUDY RESULTS**

| **Descripción**  **Description** | **Unidad**  **Units** | **Valor Basal**  **Basal** | **Valor Referencia**  **Reference Values** | **% del V.Ref Basal**  **% of RV basal** | **Valor Broncodilatador**  **Bronchodilator** | **% del V.Ref Bronco**  **% of RV bronchodilator** | **% de Cambio**  **% of change** |
| --- | --- | --- | --- | --- | --- | --- | --- |
| Cantidad Maniobras  Number of maneuvers |  | 2 |  |  | 4 |  |  |
| FVC | L | 3.6550 | 4.9725 | 73.50 | 3.7856 | 77.23 | 13 |
| FEV1 | L | 2.2806 | 3.9212 | 58.16 | 3.6044 | 92.30 | 32 |
| FEF25-75 | L/s | -9650 | 3.7012 | 88.29 | 4.4025 | 119.1 | 144 |
| PEF | L/s | 2.7512 | 7.6762 | 35.84 | 6.9731 | 89.9 | 422 |
| Fecha y hora  Data and time |  | 12/01/2012  13:30 |  |  | 12/01/2012 14:05 |  |  |
| Grado de calidad  Quality Grade |  | F |  |  | D |  |  |

### RB-S004 – SECTION FOR THE RESULTS BY MANOEUVRE

| **Restriction** | **Description** |
| --- | --- |
| ***RB-S004-***  ***01*** | The document **must** contain one section with the results of each manoeuvre. It **must** be identified with the code 'S004' of the local codification system for the sections of this document. The document **must** contain one element  /ClinicalDocument/component/structuredBody/component/section  /code/@code='S004' and @codeSystem='2.16.840.1.113883.2.19.60.2.1'  This section **must** contain the results for each maneuver, specifically for the basal test. This section **must** contain the information textually (requirements RB-S004) and coded (requirements RC-04).  In case the FS test is composed also of bronchodilator manoeuvres, it **can** contain the bronchodilator test. In this case, it **must** contain the results for each manoeuvre of the FS test, duplicating all fields of this section and identifying them with the suffix "0.1". |

| **Restriction** | **Description** |
| --- | --- |
| ***RB-S004-***  ***02*** | The section about the results of manoeuvres **must** contain the title  RESULTADOS DE LAS MANIOBRAS (Results of the manoeuvres in Spanish), '/ClinicalDocument/component/structuredBody/component/section [@code='S004']/title', in case of coding the manoeuvres from the basal test.  The section about the results of manoeuvres **must** contain the title  RESULTADOS DE LAS MANIOBRAS BRONCODILATADORAS (Results of the bronchodilator manoeuvres in Spanish), '/ClinicalDocument/component/structuredBody/component/section [@code='S004']/title', in case of coding the manoeuvres from the bronchodilator test. |

The section on the results of the manoeuvres **must** contain textually each one of the parameters marked as **mandatory** on the "Requirement" column of the following table.

The section on the results of the manoeuvres **can** contain textually each one of the parameters marked as **OPTIONAL** on the "Requirement" column of the following table.

| **Parameter** | **Description** | **Requirement** | **Unit** | **Restriction** |
| --- | --- | --- | --- | --- |
| Manoeuvre number | Manoeuvre number | Mandatory | 1 to 8 | RB-S004-03 |
| FVC | Vital Forced Capacity | Mandatory | L | RB-S004-04 |
| expected FVC | expected FVC according to the reference values used | Mandatory | L | RB-S004-05 |
| % FVC / expected FVC | Ratio FVC and expected FVC | Mandatory | % | *RB-S004-06* |
| FEV0.5 | Forced Expired Volume in ½ second | Optional | L | *RB-S004-07* |
| FEV1 | Forced Expired Volume in 1 second | Mandatory | L | *RB-S004-08* |
| expected FEV1 | FEV1 according to the reference values used | Mandatory | L | *RB-S004-09* |
| % FEV1/ expected FEV1 | Ratio FEV1 and expected FEV1 | Mandatory | % | RB-S004-10 |

| **Parameter** | **Description** | **Requirement** | **Unit** | **Restriction** |
| --- | --- | --- | --- | --- |
| FEV3 | Forced Expired Volume in 3 seconds | Optional | L | *RB-S004-11* |
| FEV6 | Forced Expired Volume in 6 seconds | Optional | L | *RB-S004-12* |
| expected FEV6 | FEV6 according to the reference tables used | Optional | L | *RB-S004-13* |
| % FEV6/expected FEV6 | Ratio FEV6 and expected FEV6 | Optional | % | *RB-S004-14* |
| % FEV0.5 / FVC | Ratio FEV0.5 and FVC | Optional | % | *RB-S004-15* |
| % FEV1 / FVC | Ratio FEV1 and FVC | Mandatory | % | *RB-S004-16* |
| % FEV3 / FVC | Ratio FEV3 and FVC | Optional | % | *RB-S004-17* |
| % FEV1 / FEV6 | Ratio FEV1 and FEV6 | Optional | % | *RB-S004-18* |
| % FEV1 / VC | Ratio FEV1 and vital capacity | Optional | % | *RB-S004-19* |
| PEF | Peak Expiratory Flow | Mandatory | L/s | *RB-S004-20* |
| expected PEF | PEF according to the reference tables used | Mandatory | L/s | *RB-S004-21* |
| % PEF / expected PEF | Ratio PEF and expected PEF | Mandatory | % | *RB-S004-22* |
| PEFT | Time needed to reach the PEF | Optional | s | *RB-S004-23* |
| FEF75% | Forced Expiratory Flow to 75% of FVC | Optional | L/s | *RB-S004-24* |
| FEF50% | Forced Expiratory Flow to 50% of FVC | Optional | L/s | *RB-S004-25* |
| expected FEF50% | FEF50% according to the reference tables used | Optional | L/s | *RB-S004-26* |
| %FEF50/expected FEF50 | Ratio FEF50 and expected FEF50 | Optional | % | *RB-S004-27* |
| FEF25% | Forced Expiratory Flow to 25% of FVC | Optional | L/s | *RB-S004-28* |
| FEF25%-75% | Mean of the Forced Expiratory Flow between 25 and 75% of FVC | Mandatory | L/s | *RB-S004-29* |
| expected FEF25%-75% | FEF25%-75% according to the reference tables used | Mandatory | L/s | *RB-S004-30* |
| % FEF25-75 / expected FEF25-75 | Ratio FEF25%-75% and expected FEF25%-75% | Mandatory | % | *RB-S004-31* |
| FET100% | Time to reach 100% | Optional | s | *RB-S004-32* |
| % FEF50% / FIF50% | Ratio between the expiratory and inspiratory flows at 50% | Optional | % | *RB-S004-33* |
| FIF50% | Inspiratory flow at 50% | Optional | L/s | *RB-S004-34* |
| expected FIF50 | FIF50 according to the reference tables used | Optional | L/s | *RB-S004-35* |
| %FIF50 / expected FIF50 | Ratio FIF50 and expected FIF50 | Optional | % | *RB-S004-36* |
| FIVC | Forced Inspired Vital Capacity | Optional | L | *RB-S004-37* |
| expected FIVC | Forced Inspired Vital Capacity according to the reference tables used | Optional | L | *RB-S004-38* |
| %FIVC / expected FIVC | Ratio FIVC and expected FIVC | Optional | % | *RB-S004-39* |
| EOTV | End of Test Volume | Optional | L | *RB-S004-40* |
| Tzero | Zero temporal point by back-extrapolation | Optional | s | *RB-S004-41* |
| BEV | Volume back-extrapolation | Optional | L | *RB-S004-42* |
| Flow/Volume curve length | Flow/Volume curve length | Optional | {tot} | *RB-S004-43* |
| Volume/Time curve length | Volume/Time curve length | Optional | {tot} | *RB-S004-44* |
| Sampling frequency | Sampling frequency | Optional | Hz | *RB-S004-45* |
| Date and Time | Date and time of the manoeuvre  execution | Optional | YYYYM  MDDHH MM | *RB-S004-46* |

**Example visualisation of section S004**

**RESULTS EACH MANOEUVRE**

| **Variable** | **Units** | **Manoeuvre** | **Manoeuvre** | **Manoeuvre** |  |
| --- | --- | --- | --- | --- | --- |
| Number |  | 1 | 2 | 3 |  |
| Forced Vital Capacity FVC | L | 5,87 | 5,55 | 5,53 |  |
| FVC Reference | L | 4,98 | 4,98 | 4,98 |  |
| %FVC/FVC Reference | % | 118 | 111 | 111 |  |
| FEV0.5 | L | 2,89 | 2,70 | 2,35 |  |
| FEV1 | L | 3,86 | 3,50 | 3,35 |  |
| FEV1 reference | L | 4,25 | 4,25 | 4,25 |  |
| %FEV1/FEV1 reference | % | 91 | 82 | 79 |  |
| FEV3 | L | 4,20 | 4,10 | 3,99 |  |
| FEV6 | L | 5,30 | 5,10 | 4,40 |  |
| FEV6 reference | L | 5,50 | 5,50 | 5,50 |  |
| % FEV6 / FEV6 reference | % | 96 | 92 | 80 |  |
| % FEV0.5 / FVC | % | 49 | 92 | 68 |  |
| % FEV1/FVC | % | 66 | 63 | 61 |  |
| % FEV3/FVC | % | 72 | 74 | 72 |  |
| % FEV1/FEV6 | % | 73 | 69 | 76 |  |
| % FEV1/VC | % | 188 | 325 | 434 |  |
| PEF | L/s | 0,67 | 0,73 | 0,78 |  |
| PEF reference | L/s | 0,80 | 0,80 | 0,80 |  |
| % PEF / PEF reference | % | 73 | 75 | 76 |  |
| PEFT | s | 17 | 25 | 23 |  |
| FEF75% | L/s | 1,31 | 1,32 | 1,33 |  |
| FEF50% | L/s | 1,41 | 1,38 | 1,32 |  |
| FEF50% reference | L/s | 1,50 | 1,50 | 1,50 |  |
| % FEF50 / FEF50 reference | % | 75 | 73 | 72 |  |

| **Variable** | **Units** | | **Manoeuvre** | | **Manoeuvre** | | **Manoeuvre** | |  |
| --- | --- | --- | --- | --- | --- | --- | --- | --- | --- |
| FEF25% | L/s | | 1,32 | | 1,45 | | 1,52 | |  |
| FEF25%-75% | L/s | | 2,32 | | 2,55 | | 2,64 | |  |
| FEF25-75 reference | L/s | | 2,40 | | 2,40 | | 2,40 | |  |
| % FEF25-75 / FEF25-75  reference |  | | | | | | | |  |
|  | % | | 93 | | 109 | | 120 | |  |
|  |  |  |  |  |  |  |  |  |  |
| FET100% |  | s | | 1,9300 | | 5,4600 | | 3,22 | |
| % FEF50% / FIF50% | % | | 75 | | 78 | | 82 | |  |
| FIF50% | L/s | | 1,31 | | 1,18 | | 1,25 | |  |
| FIF50 reference | L/s | | 1,50 | | 1,50 | | 1,50 | |  |
| % FIF50 / FIF50 reference | % | | 74 | | 80 | | 82 | |  |
| FIVC | L | | 2,31 | | 2,44 | | 2,23 | |  |
| FIVC reference | L | | 2,50 | | 3,50 | | 3,50 | |  |
| % FIFC / FIFC reference | % | | 80 | | 82 | | 85 | |  |
| EOTV | L | | 8,23 | | 7,40 | | 8,50 | |  |
| Tzero | s | | 10 | | 23 | | 17 | |  |
| BEV | L | | 1,32 | | 2,32 | | 3,20 | |  |
| Fow-volume curve longitude | {tot} | | 18 | | 25 | | 33 | |  |
| Volume-time curve longitude Curva volumen / tiempo |  | | | | | | | |  |
|  | {tot} | | 29 | | 33 | | 41 | |  |
|  |  |  |  |  |  |  |  |  |  |
| Sample rate | Hz | | 75 | | 78 | | 80 | |  |
|  | | | 12/01/20  12 13:30 | | 12/01/20  12 14:20 | | 12/01/20  12 14:30 | |  |
| Data and time |  | |  |  |  |  |  |  |  |
|  |  |  |  |  |  |  |  |  |  |

### RB-S005 – SECTION FOR THE FS SIGNALS

| **Restriction** | **Description** |
| --- | --- |
| ***RB-S005-01*** | The document **must** contain one section to represent the flow-volume graphs of the acquired FS signals. This section must be identified with the code 'S005' of the local codification system for the sections of this document. The document **must** contain one element  /ClinicalDocument/component/structuredBody/component/section  /code/@code='S005' and @codeSystem='2.16.840.1.113883.2.19.60.2.1' |

| **Restriction** | **Description** |
| --- | --- |
| ***RB-S005-***  ***02*** | The section **must** contain the title “GRÁFICO FLUJO-VOLUMEN” (Flow-Volume Graph in Spanish)  '/ClinicalDocument/component/structuredBody/component/section[@co de='S005']/title |

| **Restriction** | **Description** |
| --- | --- |
| ***RB-S005-03*** | The section **must** contain one reference to a multimedia object (a base64 JPG) representing the flow-volume graph of the FS test.  This multimedia object **must** be expressed through a coded entry – see RC-05.01. |

| **Restriction** | **Description** |
| --- | --- |
| ***RB-S005-04*** | The document **must** contain one section for the data point of the flow-volume graph. It **must** be identified with the code 'S005' of the local codification system for the sections of this document. It **must** be represented with the element  /ClinicalDocument/component/structuredBody/component/section  /code/@code='S005' and @codeSystem='2.16.840.1.113883.2.19.60.2.1' |

| **Restriction** | **Description** |
| --- | --- |
| ***RB-S005-05*** | The document **must** contain one section to represent the volume-time graphs of the acquired FS signals. This section must be identified with the code 'S005' of the local codification system for the sections of this document. The document **must** contain one element  /ClinicalDocument/component/structuredBody/component/section  /code/@code='S005' and @codeSystem='2.16.840.1.113883.2.19.60.2.1' |

| **Restriction** | **Description** |
| --- | --- |
| ***RB-S005-06*** | The section **must** contain the title “GRÁFICO VOLUMEN-TIEMPO” (Volume-Time Graph in Spanish)  '/ClinicalDocument/component/structuredBody/component/  section[@code='S005']/title' |

| **Restriction** | **Description** |
| --- | --- |
| ***RB-S005-07*** | The section **must** contain one reference to a multimedia object (a base64 JPG) representing the volume-time graph of the FS test.  This multimedia object **must** be expressed through a coded entry – see RC-05.02. |

| **Restriction** | **Description** |
| --- | --- |
| ***RB-S005-08*** | The document **must** contain one section for the data point of the volume-time graph. It **must** be identified with the code 'S005' of the local codification system for the sections of this document. It **must** be represented with the element  /ClinicalDocument/component/structuredBody/component/section/code/@code='S005' and @codeSystem='2.16.840.1.113883.2.19.60.2.1' |

#### Example of a fragment for the inclusion of a multimedia object in the body

*<component>*

*<section>*

*<code code="S005" codeSystem="2.16.840.1.113883.2.19.60.2.1"*

*displayName="Gráfico de las señales para la grafica flujo/volumen"> </code>*

*<title>GRAFICA FLUJO - VOLUMEN</title>*

*<text>*

*</text>*

*<entry>*

*<renderMultiMedia referencedObject="SIGNAL">*

*<caption>DIAGRAMA</caption>*

*</renderMultiMedia>*

*<observationMedia classCode="DGIMG" moodCode="EVN" ID="SIGNAL">*

*<templateId root='2.16.840.1.113883.2.19.60.2.6' extension='T05'/>*

*<value mediaType="image/jpeg" representation="B64">*

*"Representation of the image in B64"*

*</value>*

*</observationMedia>*

*</entry>*

*<!-- RC-06.02 / Description with the data points of the signals -->*

*...*

*</section>*

*</component>*

**Example visualisation of section S005 (in Spanish)**

**FLOW-VOLUME GRAPH VOLUME-TIME GRAPH**

#### GRAFICA FLUJO-VOLUMEN GRAFICA VOLUMEN-TIEMPO


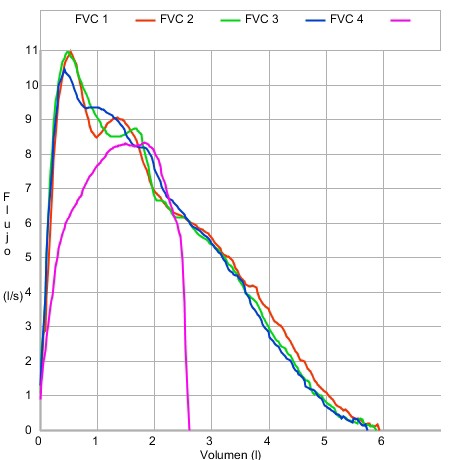

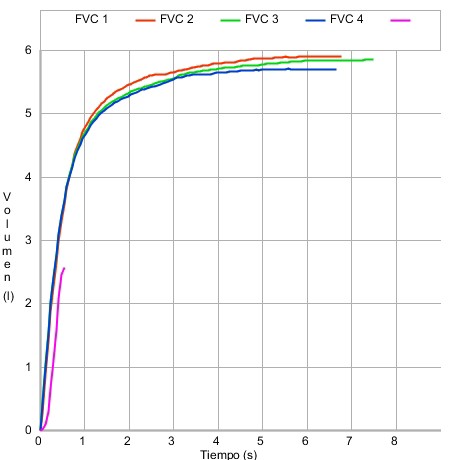


### RB-S006 – SECTION FOR COMMENTS

| **Restriction** | **Description** |
| --- | --- |
| ***RB-S006-***  ***01*** | The document **can** contain a section to specify comments. It must be identified with the code ‘S006’ of the local codification system for the sections of this document. The document **can** contain one element  /ClinicalDocument/component/structuredBody/component/section  /code/@code='S006' and @codeSystem='2.16.840.1.113883.2.19.60.2.1' |

| **Restriction** | **Description** |
| --- | --- |
| ***RB-S006-***  ***02*** | The section **must** contain a title “COMENTARIO SOBRE EL ESTUDIO” (FS test comments in Spanish)  '/ClinicalDocument/component/structuredBody/component/section [@code='S006']/title |

| **Restriction** | **Description** |
| --- | --- |
| ***RB-S006-***  ***03*** | The section **must** contain a text with comments or notes about the FS test. |

### RB-S007 – LINK TO THE GRAPHS

| **Restriction** | **Description** |
| --- | --- |
| ***RB-S007-***  ***01*** | The document **can** contain a section to represent the link to the generated graphs. In this section exists, it **must** be identified with the code ‘S007’ of the local codification system for the sections of this document. The document **must** contain one element  /ClinicalDocument/component/structuredBody/component/section/code/@code='S007' and @codeSystem='2.16.840.1.113883.2.19.60.2.1' |
| ***RB-S007-***  ***02*** | The section **must** contain the title “LINK PARA LAS GRÁFICAS” (Link to the graphs in Spanish)  '/ClinicalDocument/component/structuredBody/component/section[  @code='S007']/title |
| ***RB-S007-***  ***03*** | The section **must** contain a “text” xml tag that must include a “linkHtml” xml tag with the text of the link as content and the link reference as the value of the attribute “href”. |

**Example of the links to the graphs section**

*<section>*

*<code code=”S007” codeSystem=”2.16.840.1.113883.2.19.60.2.1” displayName=”Enlace para las gráficas”>*

*<title>LINK PARA LAS GRÁFICAS</title>*

*<text>*

*<linkHtml href=”URL”>Click aquí en caso de no visualizar las gráficas</linkHtml>*

*</text>*

*</section>*

**Example visualisation of section S007 (in Spanish)**

## RC – RESTRICTION FOR THE CODED ENTRIES

### RC-01 – PATIENT DATA SECTION

The patient data section **must** encode each one of the parameters marked as **mandatory** on the "Requirement" column of the following table.

The patient data section **can** encode each one of the parameters marked as **OPTIONAL** on the "Requirement" column of the following table.

Patient data:

| **Parameter** | **Template** | **Codes** | **Requirement** | **Unit** | **Restriction** |
| --- | --- | --- | --- | --- | --- |
| Occupation | MedSCT | SCT:14679004|Occupation | Optional |  | RC-01.1 |
| Reason for action | MedSCT | SCT: 385675009| Reason for action | Optional |  | RC-01.2 |
| Body weight | MedSCT | SCT:27113001| Body weight | Mandatory | Kg | *RC-01.3* |
| Age | MedSCT | SCT: 397669002|Age | Mandatory | Year | *RC-01.4* |
| Body height measure | MedSCT | SCT:50373000| Body height measure | Mandatory | meter | *RC-01.5* |
| Ethnic group | MedSCT | SCT:372148003| Ethnic group | Optional |  | *RC-01.6* |
| Smoker | MedSCT | SCT:77176002| Smoker | Optional |  | *RC-01.7* |

### RC-02 – SECTION FOR THE CONTEXT OF THE FS TEST

The section about the spirometer data **must** encode each one of the parameters marked as **mandatory** on the "Requirement" column of the following table.

The section about the spirometer data **can** encode each one of the parameters marked as **OPTIONAL** on the "Requirement" column of the following table.

Spirometer data:

| **Parameter** | **Template** | **Codes** | **Requirement** | **Unit** | **Restriction** |
| --- | --- | --- | --- | --- | --- |
| Transductor type | CodLoc | Code: TT (2.16.840.1.113883.2.19.60.2.5)  Value set: 2.16.840.1.113883.2.19.60.2.2 | Mandatory |  | RC-02.1 |
| Calibration Data and Time | MedSCT | SCT:118575009| Date AND time of day | Mandatory |  | RC-02.2 |
| Ambient temperature | MedSCT | SCT:250825003| Ambient temperature | Optional | ºC | *RC-02.3* |
| Atmospheric pressure | MedSCT | SCT:40513000| Atmospheric pressure | Optional | mm Hg | *RC-02.4* |
| Relative humidity | MedSCT | SCT:250829009| Relative humidity | Optional | % | *RC-02.5* |
| Reference  table used | CodLoc | Code: TR (2.16.840.1.113883.2.19.60.2.5)  Value set: 2.16.840.1.113883.2.19.60.2.7 | Optional |  | *RC-02.6* |

### RC-03 – SECTION FOR THE RESULTS OF THE FS TEST

The section about the results of the FS test **must** encode each one of the parameters marked as **mandatory** on the "Requirement" column of the following table.

The section about the results of the FS test **can** encode each one of the parameters marked as **OPTIONAL** on the "Requirement" column of the following table.

The first parameter of the study (total amount of manoeuvres) is an independent entrance.

In the case of the basal test, there is only one entry for the number of manoeuvres, with the TMFVC encoding.

For the bronchodilator test, there is a second independent entry, with the TMFVCMB encoding.

The rest of parameters **must** be grouped into a single results organizer of the type *organizer.*

For the basal test, an organizer **must** exist with the identifier code "MMFVC" | “Datos de la Mejor maniobra de la prueba basal” ("Data of the best manoeuver of the basal test" in Spanish) in the local encoding system of this guide. The parameters described in the restrictions RC-03.02 to RC-03.16 have to be included as *component* elements of this organizer, due to the fact of belonging to the best maneuver.

For the bronchodilator test, an organizer **must** exist with the identifier code "MMFVCMB" | “Datos de la Mejor maniobra de la prueba broncodilatadora” ("Data of the best manoeuver of the bronchodilator test" in Spanish) in the local encoding system of this guide. The parameters described in the restrictions RC-03.02 to RC-03.14 (defined RC-03.02.01 to RC-03.14.01 for the bronchodilator test) have to be included as *component* elements of this organizer, due to the fact of belonging to the best maneuver.

Example of organizer for the results of the FS test:

*<entry>*

*<organizer classCode=”BATTERY” moodCode=”EVN”>*

*<code code="MMFVC" codeSystem="2.16.840.1.113883.2.19.60.2.5"*

*displayName="Datos de la Mejor maniobra de la prueba Basal" />*

*<statusCode code="completed"/>*

*<component>… [here goes each one of the results] </component>*

*</organizer>*

*</entry>*

| **Parameter** | | **Template** | **Codes** | **Requirement** | **Unit** | **Restriction** |
| --- | --- | --- | --- | --- | --- | --- |
| Total amount of manoeuvres | | MedLoc | TMFVC / TMFVCMB:  Total amount of basal manoeuvres /  Total amount of bronchodilator manoeuvers 2.16.840.1.113883.2.19.60.2.5 | Mandatory | {tot} | RC-03.01 |
| Best FVC  (Forced Vital Capacity) of all manoeuvres | | MedSCT | SCT:50834005| Forced vital capacity | Mandatory | L | RC-03.02 |
| Expected FVC:  Expected FVC based on the reference values | | MedSCT | SCT:310521000| Expected forced vital capacity | Mandatory | L | RC-03.03 |
| % Best FVC / expected FVC | | MedSCT | SCT:407576000| Forced vital capacity/expected forced vital capacity percent | Mandatory | % | RC-03.04 |
| Best FEV1:  Best Forced Expired Volume in 1 second | | MedSCT | SCT:59328004|Forced expired volume in the 1^st^ second | Mandatory | L | RC-03.05 |
| Expected FEV1 | | MedSCT | SCT: 310520004| Expected forced expired volume in the 1^st^ second | Mandatory | L | RC-03.06 |
| % Best FEV1 /  expected FEV1 | | MedSCT | SCT: 313223002| Percent predicted forced expired volume in th 1^st^ second | Mandatory | % | RC-03.07 |
| % Best FEV1 /  Best FVC | | MedSCT | SCT:251944000| Forced expired volume in the 1^st^ second/forced vital capacity ratio | Mandatory | % | RC-03.08 |
| FEF25%-75% | | MedSCT | SCT:251932003| Forced expiratory flow rate between 25+75% of vital capacity | Mandatory | L/s | *RC-03.09* |
| Expected FEF25%-75% | | MedLoc | FEF25%-75%R: Expected value for the flow rate between 25+75% of FVC / 2.16.840.1.113883.2.19.60.2.5 | Mandatory | L/s | *RC-03.10* |
| % FEF25%-75% / expected FEF25%-75% | MedLoc | | FEF25%-75%RP: Percent between FEF25-75 and expected FEF25-75 /  2.16.840.1.113883.2.19.60.2.5 | Mandatory | % | *RC-03.11* |
| PEF | MedSCT | | SCT:313193002| Peak expiratory flow rate | Mandatory | L/s | *RC-03.12* |
| Expected PEF | MedSCT | | SCT: 313192007| Expected peak expiratory flow rate | Mandatory | L/s | *RC-03.13* |
| % PEF/ expected PEF | MedSCT | | SCT: 401163005| Percentage of best ever peak expiratory flow rate | Mandatory | % | *RC-03.14* |
| Data and Time | MedSCT | | SCT: 118575009| Date AND time of day | Optional | YYY  YMM DDH HMM | *RC-03.15* |
| Quality control grade | CodLoc | | Code: GQC (2.16.840.1.113883.2.19.60.2.5)  Value set: 2.16.840.1.113883.2.19.60.2.8 | Optional |  | *RC-03.16* |

### RC-04 – SECTION FOR THE RESULTS BY MANOEUVRE

This section **can** appear, i.e., it is not mandatory. If it appears, it **MUST** comply with the restrictions specified in this section.

The section for the results by manoeuvre **must** encode each one of the parameters marked as **mandatory** on the "Requirement" column of the following table.

The section for the results by manoeuvre **can** encode each one of the parameters marked as **OPTIONAL** on the "Requirement" column of the following table.

Existing manoeuvres **MUST** be grouped into a single result organizer of type *organizer*. This organizer is aimed at grouping the manoeuvres of a same type of test (basal or bronchodilator). For each maneuver, an element *component* **must** exist, composed of an element *sequenceNumber* belonging to the restriction RC-04.01 (amount of manoeuvres), and an organizer comprising all observations (restrictions from RC-04.02 to RC-04.44) of the manoeuver.

In the event that there are test bronchodilator manoeuvers, an *organizer-component-organizer* structure **MUST** exist, exactly as explained above, but with the restrictions with the suffix .01 (RC-04.01.01 – RC-04.44.01).

Example of parameters organizer for the results of each manoeuver:

*<entry typeCode="DRIV">*

*<organizer classCode="BATTERY" moodCode="EVN">*

*<statusCode code="completed"/>*

*<!-- DATA FOR THE FIRST MANOEUVER -->*

*<component>*

*<sequenceNumber value="1"/>*

*<organizer classCode="BATTERY" moodCode="EVN">*

*<statusCode code="complete"/>*

*<component>*

*… [FIRST RESULT FOR MANOEUVER 1]*

*</component>*

*<component>*

*… [SECOND RESULT FOR MANOEUVER 1]*

*</component>*

*...*

*</organizer>*

*</component>*

*<!-- DATA FOR THE SECOND MANOEUVER -->*

*<component>*

*<sequenceNumber value="2"/>*

#### Results of each manoeuver

| **Parameter** | **Template** | **Codes** | **Requirement** | **Unit** | **Restriction** |
| --- | --- | --- | --- | --- | --- |
| Number of manoeuver | MedLoc | Not coded (it is expressed as a sequenceNumber element) | Mandatory | Numeric | RC-04.01 |
| FVC | MedSCT | SCT:50834005| Forced vital capacity | Mandatory | L | RC-04.02 |
| Expected FVC | MedSCT | SCT:310521000| Expected forced vital capacity | Mandatory | L | RC-04.03 |
| % FVC / expected FVC | MedSCT | SCT:407576000| Forced vital capacity/expected forced vital capacity percent | Mandatory | % | RC-04.04 |
| FEV0.5: Forced expired volume in ½ second | MedLoc | FEV0.5  2.16.840.1.113883.2.19.60.2.5 | Optional | L | RC-04.05 |
| FEV1: Forced expired volume in 1 second | MedSCT | SCT:59328004|Forced expired volume in 1 second | Mandatory | L | RC-04.06 |
| Expected FEV1 | MedSCT | SCT: 310520004| Expected forced expired volume in 1 second | Mandatory | L | RC-04.07 |
| % FEV1 / expected FEV1 | MedSCT | SCT: 313223002| Percent predicted forced expired volume in one second | Mandatory | % | RC-04.08 |
| FEV3: Forced expired volume in 3 seconds | MedLoc | FEV3  2.16.840.1.113883.2.19.60.2.5 | Optional | L | RC-04.09 |
| FEV6: Forced expired volume in 6 seconds | MedLoc | FEV6  2.16.840.1.113883.2.19.60.2.5 | Optional | L | RC-04.10 |
| Expected FEV6 | MedLoc | FEV6R  2.16.840.1.113883.2.19.60.2.5 | Optional | L | RC-04.11 |
| % FEV6 / expected FEV6 | MedLoc | FEV6/FEV6R  2.16.840.1.113883.2.19.60.2.5 | Optional | % | RC-04.12 |
| % FEV0.5 / FVC | MedLoc | FEV0.5/FVC  2.16.840.1.113883.2.19  .60.2.5 | Optional | % | RC-04.13 |
| % FEV1 / FVC | MedSCT | SCT: 251944000 | Forced expired volume in one second/forced vital capacity ratio | Mandatory | % | RC-04.14 |
| % FEV3 / FVC | MedLoc | FEV3/FVC  2.16.840.1.113883.2.19.60.2.5 | Optional | % | RC-04.15 |
| % FEV1 / FEV6 | MedLoc | FEV1/FEV6  2.16.840.1.113883.2.19.60.2.5 | Optional | % | RC-04.16 |

| **Parameter** | **Template** | **Codes** | **Requirement** | **Unit** | **Restriction** |
| --- | --- | --- | --- | --- | --- |
| % FEV1 / VC | MedSCT | SCT: 251943006 | Forced expired volume in one second/vital capacity ratio | Optional | % | RC-04.17 |
| PEF: Peak expiratory flow rate | MedSCT | SCT:18491006 | Peak expiratory flow rate | Mandatory | L/s | RC-04.18 |
| Expected PEF | MedSCT | SCT:313192007 | Expected Peak expiratory flow rate | Mandatory | L/s | RC-04.19 |
| % PEF / expected PEF | MedSCT | SCT:401199000 | Percentage of peak expiratory flow rate variability | Mandatory | % | RC-04.20 |
| PEFT: Needed time to reach PEF | MedLoc | PEFT  2.16.840.1.113883.2.19.60.2.5 | Optional | s | RC-04.21 |
| FEF75%: Maximum expiratory flow rate at 75% of FVC | MedSCT | SCT:251921003 | Maximum expiratory flow rate at 75% of vital capacity | Optional | L/s | RC-04.22 |
| FEF50%: Maximum expiratory flow rate at 50% of FVC | MedSCT | SCT:251920002 | Maximum expiratory flow rate at 50% of vital capacity | Optional | L/s | RC-04.23 |
| Expected FEF50% | MedLoc | FEF50%R  2.16.840.1.113883.2.19.60.2.5 | Optional | L/s | RC-04.24 |
| % FEF50 / expected FEF50 | MedLoc | FEF50%/FEF50%R  2.16.840.1.113883.2.19.60.2.5 | Optional | % | RC-04.25 |
| FEF25%: Maximum expiratory flow rate at 25% of FVC | MedSCT | SCT:251919008 | Maximum expiratory flow rate at 25% of vital capacity | Optional | L/s | RC-04.26 |
| FEF25%-75%: Forced expiratory flow rate between 25+75% of FVC | MedSCT | SCT:251932003 | Forced expiratory flow rate between 25+75% of vital capacity | Mandatory | L/s | RC-04.27 |
| Expected FEF25%-75% | MedLoc | FEF25%-75%R  2.16.840.1.113883.2.19.60.2.5 | Mandatory | L/s | RC-04.28 |
| % FEF25-75 / expected FEF25-75 | MedLoc | FEF25%-75%RP  2.16.840.1.113883.2.19.60.2.5 | Mandatory | % | RC-04.29 |
| FET100%: time to reach 100% | MedLoc | FET100%  2.16.840.1.113883.2.19.60.2.5 | Optional | s | RC-04.30 |
| % FEF50% / FIF50%:  Ratio between expiratory and inspiratory low at 50% of FVC | MedLoc | FEF50%/FIF50%  2.16.840.1.113883.2.19.60.2.5 | Optional | % | RC-04.31 |

| **Parameter** | **Template** | **Codes** | **Requirement** | **Unit** | **Restriction** |
| --- | --- | --- | --- | --- | --- |
| FIF50%: Maximum inspiratory flow rate at 50% of vital capacity | MedSCT | SCT:251926008 | Maximum inspiratory flow rate at 50% of vital capacity | Optional | L/s | RC-04.32 |
| Expected FIF50% | MedLoc | FIF50%R  2.16.840.1.113883.2.19.60.2.5 | Optional | L/s | RC-04.33 |
| % FIF50 / expected FIF50 | MedLoc | FIF50%/FIF50%R  2.16.840.1.113883.2.19.60.2.5 | Optional | % | RC-04.34 |
| FIVC: Forced Inspiratory vital capacity | MedSCT | SCT:251913009| Inspiratory vital capacity | Optional | L | RC-04.35 |
| Expected FIVC | MedLoc | FIVCR  2.16.840.1.113883.2.19.60.2.5 | Optional | L | RC-04.36 |
| % FIVC / expected FIVC | MedLoc | FIVC/FIVCR  2.16.840.1.113883.2.19.60.2.5 | Optional | % | RC-04.37 |
| EOTV: End of Test Volume | MedLoc | EOTV  2.16.840.1.113883.2.19.60.2.5 | Optional | L | RC-04.38 |
| Tzero: Zero temporal point by back-extrapolation | MedLoc | Tzero  2.16.840.1.113883.2.19.60.2.5 | Optional | s | RC-04.39 |
| BEV: Back-extrapolation volume | MedLoc | BEV  2.16.840.1.113883.2.19.60.2.5 | Optional | L | RC-04.40 |
| Length of the flow/volume curve | MedLoc | LCFV  2.16.840.1.113883.2.19.60.2.5 | Optional | {tot} | RC-04.41 |
| Length of the volume/time curve | MedLoc | LCVT  2.16.840.1.113883.2.19.60.2.5 | Optional | {tot} | RC-04.42 |
| Sampling frequency | MedLoc | FM  2.16.840.1.113883.2.19.60.2.5 | Optional | Hz | RC-04.43 |
| Date and Time | MedSCT | SCT: 118575009 | Date AND time of day | Optional | YYYY  MMDD  HHMM | RC-04.44 |

### RC-05 – SECTION FOR THE FS SIGNALS

1. **Signals in JPG**

Restriction **RC-05.01:** The graph of the flow-volume signals in JPG format **MUST** be added as a multimedia object using the **Multimedia** template.

Restriction **RC-05.03**: The graph of the volume-time signals in JPG format **MUST** be added as a multimedia object using the **Multimedia** template.

#### Numerical data of the signals

The numerical data of the signals of each manoeuvre, expressed as an *observation* element according to the **Signal** template, **MUST** be grouped into an observations organizer (type *organizer*) with an element *component* for each manoeuver performed. The organizer has to have the code corresponding to the type of signal and the type of manoeuvre, to indicate among SFVFVC, SFVFVCMB, SVTFVC, and SVTFVCMB.

Example of organizer for the signals of the manoeuvres.

*<entry>*

*<organizer classCode="BATTERY" moodCode="EVN">*

*<code code=”SFVFVC” codeSystem=”2.16.840.1.113883.2.19.60.2.5”*

*displayName=”Señal Flujo/Volumen para las maniobras Basales”/>*

*<statusCode code="completed"/>*

*<!-- SIGNALS FOR THE FIRST MANOEUVER -->*

*<component>*

*<sequenceNumber value="1"/>*

*… [FIRST RESULT FOR MANOEUVER 1]*

*</component>*

*<!-- SIGNALS FOR THE SECOND MANOEUVER -->*

*<component>*

*<sequenceNumber value="2"/>*

*…*

*</component>*

*</organizer>*

*</entry>*

**Restriction RC-05.02:** The data points corresponding to the signals for each manoeuvre of the flow-volume graph **MUST** be expressed according to the **Signal** template.

**Restriction RC-05.04:** The data points corresponding to the signals for each manoeuvre of the volume-time graph **MUST** be expressed according to the **Signal** template.

### RC-06 – SECTION FOR COMMENTS

In the document, a section with comments (RB-S006) **can** exist.

In case of existing, the section for comments **must** encode each one of the parameters marked as **mandatory** on the "Requirement" column of the following table.

Observations data:

| **Parameter** | **Template** | **Codes** | **Requirement** | **Unit** | **Restriction** |
| --- | --- | --- | --- | --- | --- |
| Comments | MedSCT | SCT:281296001| Result comments | Mandatory |  | RC-06.1 |

# APPENDIX

## APPENDIX I – DETAIL OF CONSIDERED OIDs

| **Description** | **OID (root)** |
| --- | --- |
| Root Spirometry | 2.16.840.1.113883.2.19.60.2 |
| Sections (Spanish Guide) | 2.16.840.1.113883.2.19.60.2.1 |
| “Type of transducer” table (Spanish Guide) | 2.16.840.1.113883.2.19.60.2.2 |
| Type of test (Spanish Guide) | 2.16.840.1.113883.2.19.60.2.4 |
| Local concepts (Spanish Guide) | 2.16.840.1.113883.2.19.60.2.5 |
| Templates (Spanish Guide) | 2.16.840.1.113883.2.19.60.2.6 |
| “Reference values” table (Spanish Guide) | 2.16.840.1.113883.2.19.60.2.7 |
| “Quality control grade” table (Spanish Guide) | 2.16.840.1.113883.2.19.60.2.8 |
| CIP (Personal Identification Code) of the Patient | 2.16.724.4.41 |
| Primary care centres | 2.16.724.4.21.5.2 |
| Hospitals | 2.16.724.4.21.5.1 |

#### OID assignment for new producers or institutions willing to use the Guide:

If a manufacturer or institution is willing to use this guide, it is needed to obtain an OID of the company, to manage the needed identifiers (e.g., list documents, identify professionals, identify hospital information systems, etc.).

There are different agencies who manage OIDs and have the possibility of assigning new OIDs. If you do not know any organism that manage OIDs, please contact HL7 SPAIN to request an OID for the company (http://www.hl7spain.org/oid.html), using the available channel to request OIDs.

The OID of the manufacturer will be of the following type:

[ROOT_OID_MANAGER_ORGANISM].[SUBBRANCH_OID_COMPANIES].X

(X will take a concrete numerical value)

For example, in case of HL7 SPAIN:

2.16.840.1.113883.2.19.5.X (2.16.840.1.113883.2.19 is the OID of HL7 SPAIN, .5 is the sub branch that HL7 SPAIN uses to assign OID to manufactures and institutions)

From this OID, the manufactures or institution will have to maintain and manage its own sub branches and OID extensions.

## APPENDIX II – LOCAL VOCABULARIES

#### 1.1.1.2.16.840.1.113883.2.19.60.2.4 - Type of test

| **Description** | **Code** |
| --- | --- |
| Forced Vital Capacity | FVC |
| Forced Vital Capacity with a bronchodilator manoeuver | FVCMB |
| Vital Capacity | VC |
| Vital Capacity with a bronchodilator manoeuver | VCMB |
| Maximum Voluntary Ventilation | MVV |
| Maximum Voluntary Ventilation with a bronchodilator manoeuver | MVVMB |

#### 1.1.2.2.16.840.1.113883.2.19.60.2.1 – Sections of the document

| **Code** | **Title of the section** |
| --- | --- |
| S001 | Datos del Paciente (Patient data in Spanish) |
| S002 | Datos del Espirómetro (Spirometer data in Spanish) |
| S003 | Resultados del Estudio (Test results in Spanish) |
| S004 | Resultados por Maniobra (Manoeuvres results in Spanish) |
| S005 | Gráficos y Valores de las Señales (Graphs and data points of the signals in Spanish) |
| S006 | Comentarios u otras observaciones (Comments and other observations in Spanish) |
| S007 | Enlace a las gráficas (Links to the graphs in Spanish) |

#### 1.1.3.2.16.840.1.113883.2.19.60.2.2 – Type of transducer

| **Code** | **Type of transducer** |
| --- | --- |
| T001 | Disc |
| T002 | Fleisch |
| T003 | Turbine |
| T004 | Lilly |
| T005 | Ultrasounds |

#### 1.1.4.2.16.840.1.113883.2.19.60.2.7 – Reference values

| **Code** | **Reference values** |
| --- | --- |
| TR001 | SEPAR |
| TR002 | ROCA |
| TR003 | ERS |
| TR004 | KNUDSON |
| TR005 | CRAPO |
| TR006 | ZAPLETAL |
| TR007 | MORRIS |
| TR008 | AUSTRIA |
| TR009 | GUTIERREZ-CHILE |
| TR010 | BRASIL |
| TR011 | POLGAR-WENG |
| TR012 | P.PADILLA-MEXICO |
| TR013 | HANKINSON |
| TR014 | A.J. CRUZ-MEJICO |
| TR015 | GOLSHAN-IRAN |
| TR016 | GLI |

#### 1.1.5.2.16.840.1.113883.2.19.60.2.8 – Quality control grade

Quality grade base don NLHEP (National Lung Health Education Program) Guidelines (N-04).

| **Code** | **Quality control grade** |
| --- | --- |
| 0 | Not computed |
| A | Very good |
| B | Good |
| C | Acceptable |
| D | Insufficient |
| F | Unacceptable |

#### 1.1.6.2.16.840.1.113883.2.19.60.2.5 – Local concepts

| **Code** | **Concept** |
| --- | --- |
| TT | Transducer type |
| TR | Reference values used |
| TMFVC | Total amount of manoeuvers basal test |
| TMFVCMB | Total amount of manoeuvers bronchodilator test |
| FM | Minimum flow |
| MMFVC | Data of the best manoeuver of the basal test |
| MMFVCMB | Data of the best manoeuver of the bronchodilator test |
| SFVFVC | Flow/Volume signal for the basal manoeuvres |
| SFVFVCMB | Flow/Volume signal for the bronchodilator manoeuvres |
| SVTFVC | Volume/Time signal for the basal manoeuvres |
| SVTFVCMB | Volume/Time signal for the bronchodilator manoeuvres |
| EP | Pulmonary age |
| GQC | Quality Control Grade |
| FEV0.5 | Forced Expired Volume in 0,5 second |
| FEV3 | Forced Expired Volume in 3 seconds |
| FEV6 | Forced Expired Volume in 6 seconds |
| FEV6R | Expected Forced Expired Volume in 6 seconds |
| FEV6/FEV6R | Percent FEV6/FEV6R. |
| FEV0.5/FVC | Percent FEV0.5/FVC |
| FEV1/FVC | Percent FEV1/FVC |
| FEV3/FVC | Percent FEV3/FVC |
| FEV1/FEV6 | Percent FEV1/FEV6 |
| PEFT | Time to reach the maximum expiratory flow |
| FEF50%R | Expected Forced Expiratory flow at 50% of FVC |
| FEF50%/FEF50%R | Percent FEF50%/FEF50%R |
| FEF25%-75%R | Expected value for the flow rate between 25+75% of FVC |
| FEF25%-75%RP | Percent between FEF25%-75% and FEF25%-75%R |
| FEF75%-85% | Flow value between 75 and 85% of FVC |
| FET25%-75% | Time to reach a flow between 25-75% of FVC |
| FET100% | Time to reach 100% of FVC |
| FEF50%/FIF50% | Percent between FEF50% and FIF50% |
| FIF50%R | Expected maximum inspiratory flow rate at 50% of VC |
| FIF50%/FIF50%R | Expected value for FIF50%/FIF50% ratio |
| FIVCR | Expected inspired vital capacity |
| FIVC/FIVCR | Percent FIVC/FIVCR |
| FIV1R | Expected FIV1 |
| FIV1/FIV1R | Expected FIV1/FIV1 ratio |
| FIV1/FIVC | Ratio FIV1/FIVC |
| FEV1/FIV1 | Ratio FEV1/FIV1 |
| PEF/PIF | Ratio PEF/PIF |
| FEV1/FEV0.5% | Ratio FEV1/FEV0.5% |
| MTT | MTT |

| EOTV | End of test volume |
| --- | --- |
| Tzero | Zero temporal point by back-extrapolation |
| BEV | Back-extrapolation volume |
| LCFV | Length of the flow/volume curve (total number of data points) |
| LCVT | Length of the volume/time curve (total number of data points) |
| FM | Sampling frequency |
| SMFVn | Signal for the manoeuver n of the flow/volume graph (n=[1..8]) |
| SMVTn | Signal for the manoeuver n of the volume/time graph (n=[1..8]) |
| INF_ESP | Spirometry test report |

#### 1.1.7.2.16.840.1.113883.2.19.60.2.6 – Templates

| **Code** | **Title of the section** |
| --- | --- |
| T00 | Template for the spirometry test report |
| T01 | **Measurements** with **SNOMED CT** (MedSCT) |
| T02 | **Measurements** with **concepts locally defined** (MedLoc) |
| T03 | **Clinical findings** coded with locally defined concepts (CodLoc) |
| T04 | Date and time stamps |
| T05 | Multimedia Objects (**Multimedia**) |
| T06 | Signals (**Signal**) |

## APPENDIX III – REFERENCES TO INTERNATIONAL VOCABULARIES

| **Vocabulary** | **Identifier of the coding system** |
| --- | --- |
| SNOMED CT | **2.16.840.1.113883.6.96** |
| HL7 ACTCODE | **2.16.840.1.113883.5.4** |
| HL7 CONFIDENTIALITY CODE | **2.16.840.1.113883.5.25** |
| HL7 GENDERCODE | **2.16.840.1.113883.5.1** |
| HL7 ETHNICITY | **2.16.840.1.113883.5.50** |

## APPENDIX IV – TEMPLATES FOR CODED ENTRIES

### MEASUREMENTS TEMPLATES

**TEMPLATE T01 – MEASUREMENTS WITH CONCEPTS DEFINED BY SNOMED (MedSCT)**

Measurements **MUST** be expressed by means of a coded entry that **MUST** at least include:

1. An element **observation MUST** exists with:
   1. Fixed structural attributes (@classCode=”OBS”, @moodCode=”EVN”). 2. The element **code MUST** exists with:
   2. The attribute **@code MUST** contain the identifier of the measured concept in SNOMED CT.
   3. The attribute **@codeSystem MUST** contain the SNOMED CT code system identifier: 2.16.840.1.113883.6.96.
2. The element **value MUST** exists with:
   1. The attribute **xsi:type =** “data_type_HL7”. (e.g., “PQ”)
   2. The attribute **@value** with the result of the measurement.

3.3. The attribute **@unit** with the unit from the Unified Code for Units of Measure (UCUM).

4. The element **statusCode MUST** exist with the attribute **@code='completed'.**

5. The element **templateId MUST** exist with the attribute **@root= '**2.16.840.1.113883.2.19.60.2.6' and **@extension='T01'.**

#### Generic template for observations with concepts in SNOMED CT:

<observation classCode="OBS" moodCode="EVN">

<templateId root='2.16.840.1.113883.2.19.60.2.6' extension='T01' **/>**

<code code=" **identifier of the measured concept in SNOMED CT**"

codeSystem="2.16.840.1.113883.6.96"

displayName=" **SNOMED CT DESCRIPTOR**"/>

<statusCode code="completed"/>

<value xsi:type="**data_type_HL7**" value="**measurement result**" unit="**UCUM unit**"/>

</observation>

#### Usage example:

*<observation classCode="OBS" moodCode="EVN">*

*<templateId root='2.16.840.1.113883.2.19.60.2.6' extension='T01' />*

*<code code="27113001" codeSystem="2.16.840.1.113883.6.96" displayName="Body weight"/>*

*<statusCode code="completed"/>*

*<value xsi:type="PQ" value="85.0" unit="kg"/>*

*</observation>*

**Note 1:** If it is an element with xsi:type = "ST", the attributes @value and @unit of the element *value* will not be present, and the value would be indicated within the *value* element. Example:

*<observation classCode="OBS" moodCode="EVN">*

*<templateId root="2.16.840.1.113883.2.19.60.2.6" extension="T01"/>*

*<code code="27113001" codeSystem="2.16.840.1.113883.6.96" displayName="Profesión">*

*</code>*

*<statusCode code="completed"/>*

***<value xsi:type="ST">Example text</value>***

*</observation>*

**Nota 2:** If it is an element with xsi:type = "BL", the attribute @unit of the element *value* will not be present, and the attribute @value **must** take one of the following values: true / false. Example:

*<observation classCode="OBS" moodCode="EVN">*

*<templateId root="2.16.840.1.113883.2.19.60.2.6" extension="T01"/>*

*<code code="77176002" codeSystem="2.16.840.1.113883.6.96" displayName="Fumador"/>*

*<statusCode code="completed"/>*

***<value xsi:type="BL" value="false"/>***

*</observation>*

**Nota 3:** If it is an element with xsi:type = "INT", the attributes @unit of the element *value* will not be present. Example:

*<observation classCode="OBS" moodCode="EVN">*

*<templateId root="2.16.840.1.113883.2.19.60.2.6" extension="T02" />*

*<code code="TMFVC" codeSystem="2.16.840.1.113883.2.19.60.2.5" displayName="Número total maniobras Prueba Basal"/>*

*<statusCode code="completed"/>*

***<value xsi:type="INT" value="6"/>***

*</observation>*

#### TEMPLATE T02 – MEASUREMENT WITH LOCALLY DEFINED CONCEPTS (MedLoc)

Measurements **MUST** be expressed by means of a coded entry that **MUST** at least include:

1. An element **observation MUST** exists with:
   1. Fixed structural attributes (@classCode=”OBS”, @moodCode=”EVN”). 2. The element **code MUST** exists with:
   2. The attribute **@code MUST** contain the identifier of the measured concept in the local codification system.
   3. The attribute **@codeSystem MUST** contain the local codification system identifier: 2.16.840.1.113883.2.19.60.2.5.
2. The element **value MUST** exists with:
   1. The attribute **xsi:type =** “data_type_HL7”. (e.g., “PQ”)
   2. The attribute **@value** with the result of the measurement.

3.3. The attribute **@unit** with the unit from the Unified Code for Units of Measure (UCUM).

4. The element **statusCode MUST** exist with the attribute **@code='completed'.**

5. The element **templateId MUST** exist with the attribute **@root= '**2.16.840.1.113883.2.19.60.2.6' and **@extension='T02'.**

#### Generic template for observations with concepts in locally defined:

<observation classCode="OBS" moodCode="EVN">

#### <templateId root='2.16.840.1.113883.2.19.60.2.6' extension='T02' />

<code code="**identificador_de_concepto_local**"

codeSystem="2.16.840.1.113883.2.19.60.2.5"

displayName="**LOCAL DESCRIPTOR**" />

<statusCode code="completed"/>

<value xsi:type=" **data_type_HL7**" value=" **measurement result**" unit="**UCUM unit**"/>

</observation>

#### Usage example:

*<observation classCode="OBS" moodCode="EVN">*

*<templateId root='2.16.840.1.113883.2.19.60.2.6' extension='T02' />*

*<code code="EP" codeSystem="2.16.840.1.113883.2.19.60.2.5" displayName="Edad Pulmonar" />*

*<statusCode code="completed"/>*

*<value xsi:type="PQ" value="57" unit="year"/>*

*</observation>*

### 4.4.2. TEMPLATES FOR ANSWERS

#### TEMPLATE T03 – FOR LOCALLY ENCODED ANSWERS (CodLoc)

Answers **MUST** be expressed by means of a coded entry that **MUST** at least include:

1. An element **observation MUST** exists with:
   1. Fixed structural attributes (@classCode=”OBS”, @moodCode=”EVN”).

1.2. The attribute **@negationInd=true CAN** be included only in case of a negative answer (example: ASTHMA=NO).

2. The element **code MUST** exists with:

- 1. The attribute **@code MUST** contain the identifier of the measured concept in the local codification system.
  2. The attribute **@codeSystem MUST** contain the local codification system identifier: 2.16.840.1.113883.2.19.60.2.5.

1. The element **value MUST** exists with:
   1. The attribute **xsi:type =** “CD”.
   2. The attribute **@code** in the concept defined in the local table.

3.3. The attribute **@codeSystem MUST** contain the identifier for the local table.

4. The element **statusCode MUST** exist with the attribute **@code='completed'.**

5. The element **templateId MUST** exist with the attribute **@root= '**2.16.840.1.113883.2.19.60.2.6' and **@extension='T03'.**

#### Generic template for locally encoded answers:

<observation classCode="OBS" moodCode="EVN">

#### <templateId root='2.16.840.1.113883.2.19.60.2.6' extension='T03'/>

<code code="**[local_code]**" codeSystem="**[local_coding_system]**"/>

<statusCode code="completed"/>

<value xsi:type="CD"

code="**[local_value_code]**"

codeSystem="**[local_codification_Cystem_code]**"

displayName="**[description_local_code]**"/>

</observation>

#### Usage example:

*<observation classCode="OBS" moodCode="EVN">*

*<templateId root='2.16.840.1.113883.2.19.60.2.6' extension='T03' />*

*<code code="TT" displayName="Transductor type" codeSystem="2.16.840.1.113883.2.19.60.2.5" />*

*<statusCode code="completed"/>*

*<value xsi:type="CD" code="T002" codeSystem="2.16.840.1.113883.2.19.60.2.2" displayName="Fleisch"/>*

*</observation>*

#### TEMPLATE T04 – TO EXPRESS DATE AND TIME STAMPS

Answers **MUST** be expressed by means of a coded entry that **MUST** at least include:

1. An element **observation MUST** exists with:
   1. Fixed structural attributes (@classCode=”OBS”, @moodCode=”EVN”). 2. The element **code MUST** exists with:
   2. The attribute **@code MUST** contain the identifier of the measured concept in SNOMED CT or the local codification system.
   3. The attribute **@codeSystem MUST** contain the SNOMED CT code system identifier: 2.16.840.1.113883.6.96 or the local codification system identifier.
2. The element **value MUST** exists with:
   1. The attribute **xsi:type =** “TS”
   2. The attribute **@value** with the date and time stamp formatted as YYYYMMDDHHMM.

3.3. The attribute **@unit** with the unit from the Unified Code for Units of Measure (UCUM).

4. The element **statusCode MUST** exist with the attribute **@code='completed'.**

5. The element **templateId MUST** exist with the attribute **@root= '**2.16.840.1.113883.2.19.60.2.6' and **@extension='T04'.**

#### Generic template for locally coded Dates and Time stamps:

<observation classCode="OBS" moodCode="EVN">

#### <templateId root='2.16.840.1.113883.2.19.60.2.6' extension='T04' />

<code code="**[code]**" codeSystem="**[coding_system]**" />

<statusCode code="completed"/>

<value xsi:type="TS" value="**[YYYYMMDDHHMM]**"/>

</observation>

#### Usage example:

*<observation classCode="OBS" moodCode="EVN">*

*<templateId root='2.16.840.1.113883.2.19.60.2.6' extension='T04' />*

*<code code="FC" codeSystem="2.16.840.1.113883.2.19.60.2.5"/>*

*<statusCode code="completed"/>*

*<value xsi:type="TS" value="201002031030"/>*

*</observation>*

#### TEMPLATE T05 – TO EXPRESS MULTIMEDIA OBJECTS (Multimedia)

Answers **MUST** be expressed by means of a coded entry that **MUST** at least include:

1. An element **observationMedia MUST** exists with:
   1. Fixed structural attributes (@classCode=”DGIMG”, @moodCode=”EVN”, @ID=”SIGNAL”).
   2. The attribute **@ID** have to coincide with the tag for inclusion of multimedia objects (renderMultimedia) in the body of the document.

2. The element **value MUST** exists with:

- 1. The attribute **@ mediaType MUST** contain the fixed value “image/jpeg”.
  2. The attribute **@ representation MUST** contain the fixed value “B64” and **MUST** contain as value the jpeg image coded in Base64.

3. The element **templateId MUST** exist with the attribute **@root= '**2.16.840.1.113883.2.19.60.2.6' and **@extension='T05'.**

#### Generic template for Multimedia Objects:

<observationMedia classCode="**DGIMG**" moodCode="EVN" ID="**SIGNAL**">

<templateId root='2.16.840.1.113883.2.19.60.2.6' extension='**T05**' />

<value mediaType="**image/jpeg**" representation=”**B64**”>

<!-- Here jpeg image in Base64 -->

</value>

</observationMedia>

#### Usage example:

*<observationMedia classCode="DGIMG" moodCode="EVN" ID="SIGNAL">*

*<templateId root='2.16.840.1.113883.2.19.60.2.6' extension='T05' />*

*<value mediaType="image/jpeg" representation=”B64”> DFJSDKFGSJGGKLFÑMGKMBVMBIKTIP/2wLGDFKGJDKLGJ...*

*</value>*

*</observationMedia>*

#### TEMPLATE T06 – TO EXPRESS SIGNALS (Signal)

Answers **MUST** be expressed by means of a coded entry that **MUST** at least include:

1. The element **templateId MUST** exist with:

1.1. Attribute **root=”2.16.840.1.113883.2.19.60.2.6”** and **extension=”T06”.**

1. An element **code MUST** exist with:
   1. The attribute **@code MUST** contain the concept identifier SMFV or SMVT, depending on whether the signal being encoded is the corresponding to the flow-volume or volume-time graph respectively.
   2. The attribute **@codeSystem MUST** contain the identifier for the local definition table: 2.16.840.1.113883.2.19.60.2.5.
   3. The attribute **@displayName MUST** contain the value “Señal para Maniobra

<ManoeuverNumber> de la gráfica FV” (“Signal for the Manoeuver <ManoeuverNumber> of the Flow-Volume graph” in Spanish) or “Señal para Maniobra < ManoeuverNumber > de la gráfica VT” (“Signal for the Manoeuver <ManoeuverNumber> of the Volume-Time graph” in Spanish) depending on whether the signal being encoded is the corresponding to the flow-volume or volume-time graph respectively.

1. The element **statusCode MUST** exist with the attribute **@code = 'completed'.**
2. The element **value MUST** exist with the following elements:
   1. The element **origin MUST** contain the attribute **value** with the initial value – fixed to 0 (zero).
   2. The element **scale MUST** contain the attribute **value** with the value at which it is to multiply each of the integer data obtained to represent the physical value in Litters.
   3. The element **digits MUST** contain the value of each of the measurements obtained, expressed as integers.

Notes:

1. The total amount of expired litters in measurement performed **MUST** be able to be expressed as:

#### vol(i) = origin + scale * digits(i)

1. The amount of digits suggested to be provided is **256** – this implies a sampling frequency of 25 measurements per second if the total expired volume in 10 seconds were measured.
2. La variable **scale MUST** be inversely proportional to the maximum scale for measurement and proportional to the signal provided by the A/D converter.

*Example: if the maximum measured value is 10 L, to be able to provide the obtained values though integer numbers, the variable* ***scale*** *must be* ***0.01****.*

#### Generic temple for the values of the signal:

<observation classCode="OBS" moodCode="EVN">

<templateId root=”2.16.840.1.113883.2.19.60.2.6” extension='T06' />

<code="<SMFV or SMVT>"

codeSystem="2.16.840.1.113883.2.19.60.2.5"

displayName="Señal para maniobra <manoeuverNumber> de la grafica <FV or VT>"/>

<statusCode code="completed"/>

<value xsi:type="SLIST_PQ">

<origin value="**[origin]**" unit="L"/>

<scale value="**[scale]**" unit="L"/>

<digits>

#### [d1][d2][d3][d4][d5][d6][d7][d8][d9][d10] … [dn]

</digits>

</value>

</observation>

#### Usage example:

*<observation classCode="OBS" moodCode="EVN">*

*<templateId root='2.16.840.1.113883.2.19.60.2.6' extension='T06' />*

*<code="SMFV" codeSystem="2.16.840.1.113883.2.19.60.2.5" displayName="Señal para Maniobra 5 de la grafica FV"/>*

*<statusCode code="completed"/>*

*<value xsi:type="SLIST_PQ">*

*<origin value="0" unit="L"/>*

*<scale value="0.01" unit="L"/>*

*<digits>0 10 30 50 80 95 120 121 125 180 190 250 300 350 410 415 424 436 459 470 501 505 520 525 532 535 550 555 570 580 582 590 601 610 620 624 630 630 630 630 630 630 630 630 630 630 630 630 630 630 632 632 633 633 634 635 637 640 642 642 650 650 650 650 650 650 650 650 650 650 650 650 650 650 650 650 650 650 650 650 650 650 650 650 650 650 650 650 650 650 650 650 650 650 650 650 650 650 650 650 650 650 650 650 650 650 650 650 650 650 650 650 650 650 650 650 650 650 650 650 650 650 650 650 650 650 650 650 650 650 650 650 650 650 650 650 650 650 650 650 650 650 650 650 650 650 650 650 650 650 650 650 650 650 650 650 650 650 650 650 650 650 650 650 650 650 650 650 650 650 650 650 650 650 650 650 650 650 650 650 650 650 650 650 650 650 650 650 650 650 650 650 650 650 650 650 650 650 650 650 650 650 650 650 650 650 650 650 650 650 650 650 650 650 650 650 650 650 650 650 650 650 650 650 650 650 650 650 650 650 650 650 650 650 650 650 650 650 650 650 650 650 650 650 650 650 650 650 650 650 650 650 650 650 650 650*

*</digits>*

*</value>*

*</observation>*

Reference List

(1) Miller MR, Crapo R, Hankinson J, Brusasco V, Burgos F, Casaburi R et al. General considerations for lung function testing. *Eur Respir J* 2005; 26(1):153-161.

(2) Miller MR, Hankinson J, Brusasco V, Burgos F, Casaburi R, Coates A et al. Standardisation of spirometry. *Eur Respir J* 2005; 26(2):319-338.

(3) Muller-Brandes C, Kramer U, Gappa M, Seitner-Sorge G, Huls A, von BA et al. LUNOKID: can numerical American Thoracic Society/European Respiratory Society quality criteria replace visual inspection of spirometry? *Eur Respir J* 2014; 43(5):1347-1356.

(4) Burgos F, Melia U, Vallverdu M, Velickovski F, Lluch-Ariet M, Caminal P et al. Clinical decision support system to enhance quality control of spirometry using information and communication technologies. *JMIR Med Inform* 2014; 2(2):e29.

(5) Melia U, Burgos F, Vallverdu M, Velickovski F, Lluch-Ariet M, Roca J et al. Algorithm for automatic forced spirometry quality assessment: technological developments. *PLoS One* 2014; 9(12):e116238.

(6) Salas T, Rubies C, Gallego C, Munoz P, Burgos F, Escarrabill J. Technical requirements of spirometers in the strategy for guaranteeing the access to quality spirometry. *Arch Bronconeumol* 2011; 47(9):466-469.

(7) Marimon-Sunol S, Rovira-Barbera M, Acedo-Anta M, Nozal-Baldajos MA, Guanyabens-Calvet J. [Shared electronic health record in Catalonia, Spain]. *Med Clin (Barc )* 2010; 134 Suppl 1:45-48.

(8) Cano I, Alonso A, Hernandez C, Burgos F, Barberan-Garcia A, Roldan J et al. An adaptive case management system to support integrated care services: Lessons learned from the NEXES project. *J Biomed Inform* 2015; 55:11-22.

(9) Hernandez C, Alonso A, Garcia-Aymerich J, Grimsmo A, Vontetsianos T, Garcia CF et al. Integrated care services: lessons learned from the deployment of the NEXES project. *Int J Integr Care* 2015; 15:e006.

(10) Kidholm K, Ekeland AG, Jensen LK, Rasmussen J, Pedersen CD, Bowes A et al. A model for assessment of telemedicine applications: mast. *Int J Technol Assess Health Care* 2012; 28(1):44-51.
